# Efficacy evaluation of pulmonary hypertension therapy in patients with portal pulmonary hypertension: A systematic review and meta-analysis

**DOI:** 10.3389/fphar.2022.991568

**Published:** 2022-11-16

**Authors:** Ruihua Zhang, Tengfei Li, Yueming Shao, Wei Bai, Xiaoyu Wen

**Affiliations:** ^1^ Department of Hepatology, The First Hospital of Jilin University, Changchun, Jilin, China; ^2^ Center of Infectious Diseases and Pathogen Biology, The First Hospital of Jilin University, Changchun, Jilin, China; ^3^ Department of Infectious Diseases and Immunology, Shanghai Public Health Clinical Center, Fudan University, Shanghai, China; ^4^ Unit of Psychiatry, Department of Public Health and Medicinal Administration, and Institute of Translational Medicine, Faculty of Health Sciences, University of Macau, Macao, Macao SAR, China

**Keywords:** portal pulmonary hypertension, pulmonary arterial hypertension, portal hypertension, curative effect, meta-analysis

## Abstract

**Objective:** To determine the therapeutic effect of pulmonary arterial hypertension (PAH) agents for portal pulmonary hypertension (POPH).

**Design:** Systematic review and meta-analysis.

**Background:** POPH is a serious complication of end-stage liver disease with a low survival rate. Liver transplantation (LT) is an effective treatment. Due to the presence of POPH, some patients cannot undergo LT. After PAH treatment, patients with POPH can obtain good hemodynamics and cardiac function for LT, but there are no standard guidelines.

**Methods:** Two independent researchers searched PubMed, EMBASE, Cochrane Library, and Web of Science for studies published from inception to 27 September 2022, focusing on the changes in hemodynamics and cardiac function in all patients with POPH to understand the effect of PAH treatment on the entire population of POPH patients. Among these, we specifically analyzed the changes in hemodynamics and cardiac function in moderate and severe POPH patients. After collecting the relevant data, a meta-analysis was carried out using the R program meta-package.

**Results:** A total of 2,775 literatures were retrieved, and 24 literatures were included. The results showed that in all POPH patients (*n* = 1,046), the following indicators were significantly improved with PAH agents: mPAP: (MD = −9.11 mmHg, *p* < 0.0001); PVR: (MD = −239.33 dyn·s·cm^−5^, *p* < 0.0001); CO: (MD = 1.71 L/min, *p* < 0.0001); cardiac index: (MD = 0.87 L/(min·m^2^), *p* < 0.0001); 6MWD: (MD = 43.41 m, *p* < 0.0001). In patients with moderate to severe POPH (*n* = 235), the following indicators improved significantly with PAH agents: mPAP (MD = −9.63 mmHg, *p* < 0.0001); PVR (MD = −259.78 dyn·s·cm^−5^, *p* < 0.0001); CO (MD = 1.76 L/min, *p* < 0.0001); Cardiac index: (MD = 1.01 L/(min·m^2^), *p* = 0.0027); 6MWD: (MD = 61.30 m, *p* < 0.0001).

**Conclusion:** The application of PAH agents can improve cardiopulmonary hemodynamics and cardiac function in patients with POPH, especially in patients with moderate to severe POPH, and the above changes are more positive.

**Systematic Review Registration:** https://inplasy.com, identifier INPLASY202250034.

## 1 Introduction

Pulmonary arterial hypertension (PAH) is a clinical and pathophysiological syndrome of altered pulmonary vascular structure or function caused by a variety of etiologies and pathogenesis, leading to increased PVR (pulmonary vascular resistance) and pulmonary arterial pressure, which progresses to right heart failure or even death. The pathology is characterized by the proliferation of endothelial cells, smooth muscle cells, and fibroblasts in the vascular wall, leading to pulmonary artery stenosis and occlusion (Tuder et al., 2013). The increase in pulmonary vascular resistance may lead to severe PAH. Portal pulmonary hypertension (POPH) is a clinical symptom with elevated pulmonary artery pressure based on portal hypertension (with or without chronic liver disease). In 1951, Mantz and Craige (Mantz and Craige, 1951) identified the first case of POPH. The Sixth World Conference on pulmonary hypertension (Xu and Jing, 2018) in 2018 classified POPH as Group 1 PAH. According to the 2016 practice guideline of the International Society for LT (Liu and Li, 2016), POPH is graded according to mPAP (mean pulmonary artery pressure) measured by RHC (right heart catheterization), which can be classified as mild (25 mmHg ≤ mPAP <35 mmHg), moderate (35 mmHg ≤ mPAP < 45 mmHg) and severe (mPAP ≥ 45 mmHg).

The incidence of POPH varies in different studies, accounting for 5%–10% (Badesch et al., 2010) in PAH, 1%–2% (Mancuso et al., 2013) in patients with portal hypertension, and 2%–6% (Savale et al., 2017) in patients with LT. Unfortunately, the survival rate is low. The median survival time of untreated POPH has been reported to be as low as 6 months (Robalino and Moodie, 1991), with a 5-year survival rate of 14%–28% and a 5-year survival rate of 35%–45% after treatment with pulmonary vasodilators alone (Krowka et al., 2000; Bremer et al., 2007; Galiè et al., 2009; Johnson et al., 2012). Currently, LT is considered to be an attractive treatment as it can cure potential liver diseases (Safdar et al., 2012), and it has been proven that pulmonary vascular diseases may be reversible in some POPH patients who survive transplantation (Scott et al., 1993; Csete, 1997; Schott et al., 1999; Krowka et al., 2000). However, the presence of PAH was found to increase mortality and prolong the time of hospitalization for LT (Krowka et al., 2016). Patients with mild POPH have mostly survivable conditions after transplantation, while patients with moderate and severe POPH have a mortality rate as high as 50%–100% after transplantation. Due to the high perioperative mortality, moderate and severe diseases are contraindications to LT, but if patients can obtain good right heart function and hemodynamics (mPAP <35 mmHg and PVR <400 dyn·s·cm^−5^) after pulmonary vasodilator therapy, some can successfully undergo LT. Therefore, PAH treatment is essential in patients with POPH, especially in patients with moderate and severe POPH.

At present, there are no formal guidelines on the clinical management of POPH. Because POPH is pathologically similar to other forms of PAH, the current clinical treatment of POPH is related to the treatment of PAH. The meta-analysis we conducted aimed to complement the existing clinical studies of POPH to evaluate the effectiveness and safety of specific treatment for pulmonary hypertension in patients with portal pulmonary hypertension to provide a basis for rational clinical drug use.

## 2 Materials and methods

### 2.1 Search strategy and study selection

This meta-analysis was conducted following the recommendations of the Preferred Reporting Items for Systematic Reviews and Meta-Analyses (PRISMA) and was registered in INPLASY (registration number: INPLASY202250034; https://inplasy.com). Two researchers (RHZ and TFL) searched PubMed, EMBASE, Web of Science and the Cochrane library for clinical studies related to the application of PAH treatment in patients with POPH. The search was conducted from the time the database was created to 27 September 2022. The following terms were applied to search the title and abstracts: “portalpulmonary hypertension” or “portal pulmonary hypertension” or “POPH” or “PPHTN.”

The titles and abstracts of retrieved studies were screened independently by two researchers, who then read the full text of the potentially included studies to select those for inclusion in the meta-analysis. All selected studies needed to meet the following criteria according to the PICOS acronym: Participants (*P*): POPH patients identified by RHC and identified as POPH patients in each study; Intervention (I): Specific treatment of PAH (including prostacyclin and its analogs, endothelin receptor antagonists, phosphodiesterase 5 inhibitors, soluble guanylate cyclase stimulants, *etc.*), regardless of whether LT was performed after drug treatment; Control (C): Baseline hemodynamic (mPAP, PVR, PAWP (pulmonary arterial wedge pressure), TPG (transpulmonary gradient), SvO2 (mixed venous oxygen saturation), CO (cardiac output), Cardiac index, RAP (right atrial pressure) or cardiac function [6MWD (6-min walking distance), NYHA (New York Heart Association) grade, WHO FC (WHO functional classification), *etc.*] were available in the study; Outcome (O): There are corresponding follow-up values in the study: hemodynamics (mPAP, PVR, PAWP, TPG, SvO2, CO, cardiac index, RAP), cardiac function (6MWD, NYHA grade, WHO FC, *etc.*) and other relevant data. Study (S): RCTs, prospective studies and retrospective studies were included. Exclusion criteria included the following: 1) Studies with <5 patients with POPH; 2) On-English articles; 3) Review, comments, conference paper, guidelines, editorial, letter, note, poster, erratum, replies, short surveys, clinical trials registration, meta; 4) Pediatric research; and 5) Incomplete data. Any discrepancies in the selection process of the included studies in the meta-analysis were resolved by consensus through discussion with a third researcher (XYW).

### 2.2 Data extraction and quality assessment

The following information was independently extracted by two researchers (YMS and WB): author name, year, country, study duration, study design, sample size, mean age, severity of underlying liver disease, etiology, treatment regimen, hemodynamic indices (mPAP, PVR, PAWP, TPG, SvO2, CO, cardiac index, RAP), 6MWD, NYHA grade, WHO FC, survival rate, adverse events, *etc.*). Study quality was assessed using the NIH Quality Assessment Tool for Case Series Studies or Controlled Intervention Studies (https://www.nhlbi.nih.gov/health-topics/study-quality-assessment-tools). Quality assessment consisted of 9 parts for case series studies and 14 parts for controlled intervention studies. The results were marked as Yes, NO, Other (cannot determine, CD; not applicable, NA; not reported, NR) (Supplementary Table S1). Any discrepancies in these processes were resolved by consensus through discussion with a third researcher (XYW).

### 2.3 Statistical analyses

Meta-analysis was conducted using a meta-package (version 5.1–0) in the R program (version 4.1.1). Mean and standard deviation (SD) were used to calculate the pooled results using a random-effects model, and for studies with median and interquartile ranges, they were transformed to mean and SD to combine data. The heterogeneity of the studies was evaluated using *I* ^*2*^, and a value of *I* ^*2*^ above 50% indicated high heterogeneity. Subgroup and meta-regression analyses were performed to explore potential sources of heterogeneity. For changes in indicators with significant heterogeneity and more than 3 studies, the sources of heterogeneity were discussed according to the following categorical variables: age ≥55 and <55; sample size ≥20 and <20; proportion of women ≥50% and <50%; prostacyclin and its analogs and nonprostacyclin and its analogs (e.g., endothelin receptor antagonists and phosphodiesterase 5 inhibitors). Meta-regression analysis was performed by the following continuous variables: the effects of baseline mPAP and PVR on the changes in mPAP and PVR in all patients with POPH and patients with moderate and severe POPH. Publication bias was evaluated by funnel plots and Egger’s test. A statistically significant level was set as *p* < 0.05 (two-tailed) for all tests.

## 3 Results

### 3.1 Basic characteristics

**FIGURE 1:**
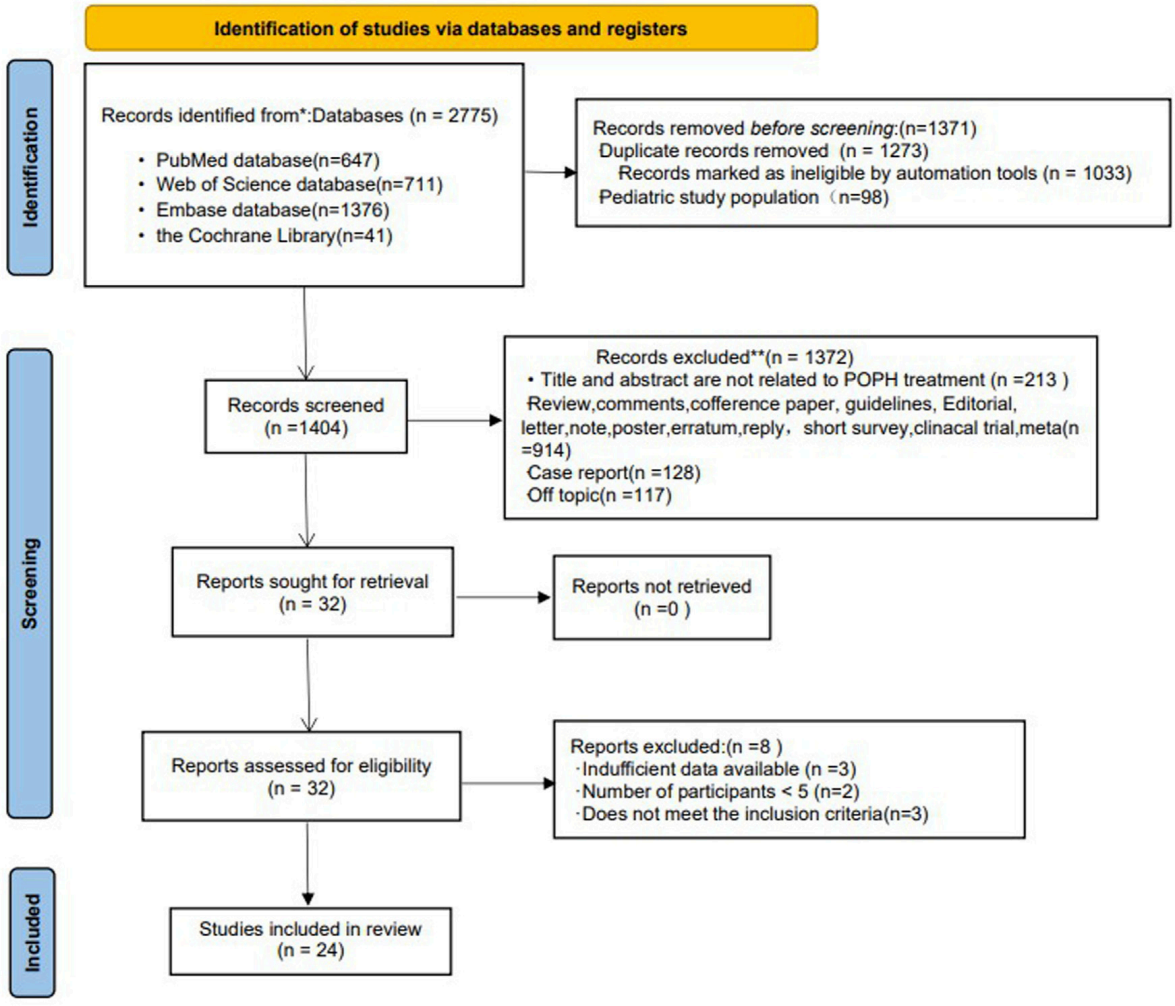
Flow chart of the selection of studies for inclusion in the meta-analysis.

Figure 1 shows the selection process of studies included in the meta-analysis. A total of 2,775 literatures were introduced into the search strategy. Of these, 24 literatures and 27 studies met the inclusion criteria (Krowka et al., 1999; Hoeper et al., 2005; Reichenberger et al., 2006; Sussman et al., 2006; Ashfaq et al., 2007; Fix et al., 2007; Hoeper et al., 2007; Gough and White, 2009; Hemnes and Robbins, 2009; Melgosa et al., 2010; Cartin-Ceba et al., 2011; Halank et al., 2011; Hollatz et al., 2012; Awdish and Cajigas, 2013; Savale et al., 2013; Khaderi et al., 2014; Fisher et al., 2015; Legros et al., 2017; Sitbon et al., 2019; DuBrock et al., 2020; Preston et al., 2020; Savale et al., 2020; Rossi et al., 2021; Sadd et al., 2021). Of these studies, 9 were in Europe [4 in Germany, 3 in France, 1 in Italy and 1 in Spain), 12 in the United States, 1 in Canada, and 1 in Europe/the United States/Brazil (the only RCT (randomized controlled trial) (Sitbon et al., 2019)] (Detail informations were shown in Table 1). Among the included studies, 3 were prospective studies (Gough and White, 2009; Cartin-Ceba et al., 2011; Awdish and Cajigas, 2013), 1 was an RCT (Sitbon et al., 2019), 1 was an open-label study (Preston et al., 2020), and the others were retrospective studies.

**TABLE 1:** Characteristics of the 27 included studies.

Author name	year	Country	Patients,n	Male (%)	Mean age (Years)	Mean MELD	Child-pugh A/B/C/- or mean points	PAH Therapy, n	PAH therapy
Rossi.R	2021	Italy	64	65.63	60	10	7.5	64	Sildenafil:38; Macitentan + Sildenafil:26
Sadd.C J**	2021	United States	24	37.5	52.8	13	NA	24	Sildenafil monotherapy:4; Sildenafil + epoprostenol:6; Sildenafil + treprostinil:5; Treprostinil:3; Sildenafil + ambrisentan:3; Sildenafil + bosentan:1; Sildenafil + inhaled iloprost:1; Ambrisentan monotherapy:1
Sadd.C J**	2021	United States	25	72	56.2	12.5	NA	25	Sildenafil:14; Sildenafil + epoprostenol:4; Sildenafil + treprostinil:5; Sildenafil + ambrisentan:1:Sildenafil + macitentan:1
DuBrock.H M**	2020	United States	16	31.25	49.5	19.5	NA	16	Sildenafil:5; Sildenafil + epoprostenol:3; epoprostenol:2; Ambrisentan + Tadalafil:1; NO:1; Ambrisentan:1; Ambrisentan + epoprostenol:1; Remodulin + sildenafil:1; Remodulin:1
Savale.L	2020	France	637	58.4	55	11.5	328/192/57/-	574	sildenafil:239; tadalafil:97; bosentan:90; ambrisentan: 36; maci-tentan: 2; bosentan + sildenafil:31; bosentan + tadalafil:15; ambrisentan + sildenafil:17; ambrisentan + tadalafil:24; macitentan + tadalafil:2
Preston.I R	2020	NA	31	41.94	62.6	11.1	13/18/−/−	23	Ambrisentan:23
Sitbon.O	2019	Europe/the United States/Brazil	43	51.16	58	8.5	20/3/-/20	43	macitentan:43
Legros.L	2017	France	20	65	52.25	11.75	8/10/2/-	20	sildenafil:10; bosentan:5; bosentan + sildenafil:3; bosentan + tadalafil:1; ambrisentan:1; associated IV epoprostenol:2
Fisher.J H	2015	Canada	20	60	54.5	15.25	3/7/3/7	20	sildenafil:19; tadalafil:1
Khaderi.S**	2014	United States	7	42.86	45	NA	NA	7	IV EPO:6; sildenafil:1
Savale.L	2013	France	34	47.06	50	NA	19/9/−/−	34	bosentan:34
Awdish.R L A	2013	United States	21	52.38	55	12.5	5/11/5/-	21	Prostacyclin IV:14; Prostacyclin inhaled:1; PDEI:5; ETRA:1
Hollatz.T J**	2012	United States	11	45.45	51.4	15.4	NA	11	Sildenafil:2; treprostinil:4; Sildenafil + epoprostenol:3; Sildenafil + inhaled iloprost:1; bosentan + Sildenafil + epoprostenol:1
Halank.M**	2011	Germany	14	35.71	55.75	10.5	12/−/−/2	14	ambrisentan + sildenafil:1; ambrisentan + tadalafil:1; ambrisentan:12
Cartin-Ceba.R**	2011	United States	13	53.85	56.5	10.88	8/−/−/5	13	ambrisentan:13
Melgosa.M T	2010	Spain	12	66.67	51	11.1	NA	12	inhaled iloprost:9; bosentan + inhaled iloprost:3; add sildenafil:1
Hemnes.A R**	2009	United States	10	50	51	14.3*	NA	10	Sildenafil:10
Gough.M S**	2009	United States	11	36.36	50.36	13.73	0/7/4/-	11	sildenafil:11
Ashfaq.M**	2007	United States	8	75	52.4	11.9	7.3	6	Epoprostenol:5; Bosentan + epoprostenol:1
Ashfaq.M**	2007	United States	12	41.67	51.8	15.2	8.9	10	Epoprostenol:8; Bosentan + diltiazem + epoprostenol:1; Diltiazem:1
Hoeper.M M**	2007	Germany	13	38.46	44	12	10/3/−/−	13	inhaled iloprost:9; bosentan + inhaled iloprost:4
Hoeper.M M**	2007	Germany	18	50	48	10	18/−/−/-	18	bosentan:16; Sildenafil + bosentan:2
Fix.O K**	2007	United States	19	63.16	49.9	15.25	6/8/5/-	19	Epoprostenol:10; Epoprostenol + Sildenafil:7; Sildenafil:2
Reichenberger**	2006	Germany	12	33.33	55	NA	7/5/−/−	12	sildenafil:7; sildenafil + inhaled iloprost:5
Sussman.N**	2006	United States	8	62.5	45.75	17.38	NA	8	Epoprostenol:8
Hoeper.M M**	2005	Germany	11	45.45	51.55	NA	11/−/−/-	11	bosentan:11
Krowka.M J**	1999	United States	7	71.43	45.43	NA	3/3/1/-	7	Epoprostenol:7

Note: PAH, Pulmonary hypertension; *:Mean MELD, in 7 patients; NA:Not available. **: 18 studies on the treatment of patients with moderate and severe POPH.

The basic characteristics of the studies are shown in Table 1. A total of 1,046 POPH patients with a mean age of 54.49 years were included in these studies. Among them, women accounted for 44.16%. The most common cause of portal hypertension was alcoholic liver disease (49.15%) (Table 2). Patients in the study received various PAH-specific treatments (Table 1), including prostacyclin and its analogs (epoprostenol, treprostinil, remodulin, inhaled iloprost), endothelin receptor antagonists (ambrisentan, macitentan, bosentan), and phosphodiesterase 5 inhibitors (sildenafil, tadalafil). There were 15 literature on the treatment of patients with moderate and severe POPH (*n* = 235), including 18 studies (Table 1).

**TABLE 2:** Characteristics of the 27 Included Studies: etiology of liver Disease.

First author	year	patients, n	ALD	Viral (HCV/HBV)	ALD + viral	Autoimmune (AIH/PBC/PSC)	Cryptogenic	Cholestatic	Metabolic	Other
Rossi.R	2021	64	11	32 (−/−)	7	0	0	0	8	6
Sadd.C J	2021	24	11	3 (3-/)	4	0	1	1	3	1
Sadd.C J	2021	25	10	4 (4/-)	6	0	2	0	2	1
DuBrock.H M	2020	16	7	7 (7/-)	0	2 (1/-/1)	2	0	1	2
Savale.L	2020	637	370	83 (−/−)	100	21 (−/−/-)	15	0	22	26
Preston.I R	2020	31	10	11	0	6 (-/4/-)	0	0	5	1
Sitbon.O	2019	43	24	9 (9/-)	3	4 (-/1/-)	0	0	2	1
Legros.L	2017	20	13	2 (2/-)	1	1 (−/−/-)	0	0	0	3
Fisher.J H	2015	20	11	2 (−/−)	0	1 (−/−/-)	0	0	2	4
Khaderi.S	2014	7	2	3 (3/-)	0	0	2	0	0	0
Savale.L	2013	28	20	4 (4/-)	3	1 (−/−/-)	0	0	0	0
Awdish.R L A	2013	21	NA	NA	NA	NA	NA	NA	NA	NA
Hollatz.T J	2012	11	5	1 (1/-)	3	0	0	1	0	1
Halank.M	2011	14	8	1 (-/1)	0	1 (-/1/-)	3	0	0	1
Cartin-Ceba.R	2011	13	6	3 (3/-)	0	1 (−/−/-)	0	0	1	2
Melgosa.M T	2010	12	2	5 (4/1)	0	2 (-/2/-)	0	0	0	4
Hemnes.A R	2009	10	0	7 (7/-)	1	1 (-/1/-)	0	0	0	1
Gough.M S	2009	11	4	2 (2/-)	4	0	0	0	0	1
Ashfaq.M	2007	8	0	3 (3/-)	0	0	1	3	0	1
Ashfaq.M	2007	12	0	4 (4/-)	0	0	3	1	0	4
Hoeper.M M	2007	13	6	2 (−/−)	0	4 (−/−/-)	0	1	0	0
Hoeper.M M	2007	18	11	3 (−/−)	0	2 (−/−/-)	0	2	0	0
Fix.O K	2007	36*	7	9 (9/-)	10	3 (-/2/-)	3	1	0	3
Reichenberger	2006	12	7	2 (−/−)	0	3 (−/−/-)	0	0	0	0
Sussman.N	2006	8	4	1 (1/-)	1	0	2	0	0	0
Hoeper.M M	2005	11	7	1 (1/-)	0	0	2	1	0	0
Krowka.M J	1999	7	0	1 (1/-)	2	3 (1/-/2)	1	0	0	0

Note: AIH, autoimmune hepatitis; ALD, alcoholic liver disease; HBV, hepatitis B virus; HCV, hepatitis C virus; PBC, primary biliary cholangitis; PSC, primary sclerosing cholangitis; *: Epoprostenol group:19 + Nonepoprostenol:17.

### 3.2 Effect of PAH-specific treatment on pulmonary hemodynamics in all patients with POPH

In all POPH patients, the following indices were significantly improved after PAH treatment: mPAP (MD = −9.11 mmHg, *p* < 0.0001); PVR (MD = −239.33 dyn·s·cm^−5^, *p* < 0.0001); PAWP (MD = 1.36 mmHg, *p* = 0.0303); TPG (MD = −13.81 mmHg, *p* < 0.0001); and SvO2 (MD = 5.16%, *p* < 0.0001). After 6 months of PAH treatment, mPAP (MD = −7.5 mmHg, *p* < 0.0001) and PVR (MD = −176.66 dyn·s·cm^−5^, *p* < 0.0001) were significantly improved, and the change in PAWP (MD = 0 mmHg, *p* = 0.9938) was not obvious. After 1 year of PAH treatment, PVR (MD = −236.54 dyn·s·cm^−5^, *p* = 0.0433) improved significantly, while mPAP (MD = −3.46 mmHg, *p* = 0.2176) did not improve significantly (Detail informations were shown in Table 3, Supplementary Figure S1).

**TABLE 3:** Changes of hemodynamics and 6 MWD in all patients with POPH.

	Number of studies	Merge results	95%*CI*	*I* ^*2*^ (%)	*p*-Value
Overall mPAP change	25	−9.11	(−10.97,−7.26)	54	<0.0001
Changes in mPAP at 6 months	3	−7.5	(−10.40,−4.59)	0	<0.0001
Changes in mPAP at 1 year	3	−3.46	(−8.97,2.04)	0	0.2176
Overall PVR change	25	−239.33	(−272.28,−206.37)	77	<0.0001
Changes in PVR at 6 months	3	−176.66	(−247.89,−105.43)	11	<0.0001
Changes in PVR at 1 year	3	−236.54	(−465.96,−7.13)	0	0.0433
Overall PAWP change	14	1.36	(0.13,2.59)	74	0.0303
Changes in PAWP at 6 months	3	0	(−1.12,1.13)	0	0.9938
Overall TPG change	5	−13.81	(−15.95,−11.67)	29	<0.0001
Overall SVO2 change	8	5.16%	(3.19%,7.14%)	38	<0.0001
Overall CO change	19	1.71	(1.28,2.14)	54	<0.0001
Overall Cardiac index change	11	0.87	(0.61,1.12)	93	<0.0001
Overall RAP change	12	−1.22	(−2.44,−0.01)	62	0.0479
Overall 6MWD change	16	43.41	(29.48,57.34)	39	<0.0001
Changes in 6MWD at 3 months	3	33.56	(−2.18,69.30)	16	0.0657
Changes in 6MWD at 6 months	4	22.97	(10.37,35.56)	0	0.0004
Changes in 6MWD at 1 year	7	66.84	(47.48,86.20)	0	<0.0001

Note: 6MWD, 6-minutes walking distance; 95%*CI*, 95%confidence interval; CO, cardiac output; mPAP, mean pulmonary artery pressure; PAWP, pulmonary wedge pressure; PVR, pulmonary vascular resistance; RAP, right atrial pressure; SvO2, mixed venous oxygen saturation; TPG, transpulmonary gradient.

### 3.3 Effects of PAH-specific treatment on cardiac hemodynamics and cardiac function of all patients with POPH

In all POPH patients, the following indices were significantly improved after PAH treatment: CO: (MD = 1.71 L/min, *p* < 0.0001); cardiac index: (MD = 0.87 L/(min·m^2^), *p* < 0.0001); RAP (MD = −1.22 mmHg, *p* = 0.0479); 6MWD (MD = 43.41 m, *p* < 0.0001). Three studies (Hollatz et al., 2012; Preston et al., 2020; Rossi et al., 2021) reported changes in right ventricular function or size. Hollatz et al. (2012) followed up eight patients and found no change in right ventricular size in two patients. The expansion degree of five patients was reduced, and that of one patient was increased. In right ventricular function, six patients improved, and two remained unchanged. Preston et al. (2020) followed patients and found reduced right ventricular enlargement in six patients and enlargement in three patients. In terms of right ventricular function, eight patients improved, and two worsened. Rossi et al. (2021) found that after 6 months of sildenafil application, overall RV performance improved significantly in patients with POPH, with significant increases in RV volume (+33%), RV ejection fraction (+31%), and RV work index (+17.5%). After PAH treatment, except for the unclear change in 6MWD at 3 months (MD = 33.56 m, *p* = 0.0657), 6MWD at 6 months (MD = 22.97 m, *p* = 0.0004) and 6MWD at 1 year (MD = 66.84 m, *p* < 0.0001) were significantly improved. Four studies (Hoeper et al., 2005; Hoeper et al., 2007; Swanson et al., 2008; Savale et al., 2013) showed that the proportion of patients with NYHA grade III/IV decreased after PAH treatment. Three studies (Gough and White, 2009; Fisher et al., 2015; Preston et al., 2020) reported a decrease in the proportion of WHO FC III/IV patients (Detail informations were shown in Table 3, Supplementary Figure S1).

### 3.4 Effects of PAH-specific treatment on cardiopulmonary hemodynamics and 6MWD of patients with moderate and severe POPH

In patients with moderate to severe POPH, except for the insignificant improvement in SvO2 (MD = 3.36%, *p* = 0.1714) and RAP (MD = −0.53 mmHg, *p* = 0.6473), the following indices were significantly improved after PAH treatment: mPAP (MD = −9.63 mmHg, *p* < 0.0001); PVR (MD = −259.78 dyn·s·cm^−5^, *p* < 0.0001); PAWP (MD = 2.45 mmHg, *p* = 0.0217); TPG (MD = −14.86 mmHg, *p* < 0.0001); CO (MD = 1.76 L/min, *p* < 0.0001); Cardiac index (MD = 1.01 L/(min·m^2^), *p* = 0.0027); 6MWD (MD = 61.30 m, *p* < 0.0001). The 6MWD (MD = 66.67 m, *p* < 0.0001) also showed significant improvement after 1 year of PAH treatment (Detail informations were shown in Table 4, Supplementary Figure S2).

**TABLE 4:** Changes of hemodynamics and 6MWD in patients with moderate and severe POPH.

	Number of studies	Merge results	95%*CI*	*I* ^*2*^ (%)	*p*-Value
Overall mPAP change	17	−9.63	(−12.49,−6.78)	62	<0.0001
Overall PVR change	17	−259.78	(−301.56,−218.01)	23	<0.0001
Overall PAWPchange	7	2.45	(0.36,4.54)	76	0.0217
Overall TPG change	4	−14.86	(−16.23,−13.50)	0	<0.0001
Overall SVO2 change	4	3.36%	(−1.46%,8.19%)	30	0.1714
Overall CO change	15	1.76	(1.16,2.36)	58	<0.0001
Overall Cardiac index change	4	1.01	(0.35,1.67)	83	0.0027
Overall RAP change	6	−0.53	(−2.83,1.76)	57	0.6473
Overall 6MWD change	8	61.3	(41.38,81.21)	0	<0.0001
Changes in 6MWD at 1 year	7	66.67	(38.58,94.76)	0	<0.0001

Note: 6MWD, 6-minutes walking distance; 95%*CI*, 95%confidence interval; CO, cardiac output; mPAP, mean pulmonary artery pressure; PAWP, pulmonary wedge pressure; PVR, pulmonary vascular resistance; RAP, right atrial pressure; SvO2, mixed venous oxygen saturation; TPG, transpulmonary gradient.

### 3.5 Effect of PAH treatment on the survival rate of patients with POPH

The survival rates of POPH from the date of diagnosis of POPH were as follows (Sadd et al., 2021): after PAH treatment, the 1-year survival rate was 74.5%, the 3-year survival rate was 59.3%, and the 5-year survival rate was 35.9%; after PAH combined with LT, the 1-year survival rate was 95.8%, the 3-year survival rate was 90.9%, and the 5-year survival rate was 90.9%.

From the date of PAH treatment, the survival rates for POPH were as follows: the 6-month survival rate after PAH treatment alone (Melgosa et al., 2010) was 91%, and three studies (Hoeper et al., 2007; Melgosa et al., 2010)showed that the 1-year survival rates were 77%, 83% and 94%, respectively. Two studies (Hoeper et al., 2007) showed that the 2-year survival rates were 62% and 89%, respectively, and two studies (Hoeper et al., 2007) showed that the 3-year survival rates were 46% and 89%, respectively.

Starting from the date of LT, three studies (Ashfaq et al., 2007; DuBrock et al., 2020; Sadd et al., 2021) eported 1-year survival rates of 69%, 86.9% and 90.9%, respectively, one study (Ashfaq et al., 2007) showed a 2-year survival rate of 80.8%, two studies (DuBrock et al., 2020; Sadd et al., 2021) showed 3-year survival rates of 53.8% and 86.9%, and three studies (Ashfaq et al., 2007; DuBrock et al., 2020; Sadd et al., 2021) showed 5-year survival rates of 53.8%, 67.63% and 86.9%, respectively.

### 3.6 Stop PAH treatment

After PAH combined with LT, twelve studies reported that the proportion of successful cessation of PAH treatment after LT (*n* = 77) was 53.25% (*n* = 41), and one study (Khaderi et al., 2014) had a recurrence of POPH after LT.

### 3.7 Adverse reactions after PAH treatment

A total of 12 studies reported adverse reactions after medication, and four studies (Krowka et al., 1999; Sussman et al., 2006; Ashfaq et al., 2007; Hoeper et al., 2007) noted adverse reactions after prostacyclin application. Six studies (Hoeper et al., 2007; Cartin-Ceba et al., 2011; Halank et al., 2011; Legros et al., 2017; Sitbon et al., 2019; Preston et al., 2020) reported adverse reactions after the application of endothelin receptor antagonists. One study (Fisher et al., 2015) reported adverse reactions after the application of PDE-5 inhibitors.

### 3.8 Subgroup analysis

The results showed that PAH treatment could improve cardiopulmonary hemodynamics and cardiac function, but there was significant heterogeneity (*I* ^*2*^ > 50%) in the changes in overall mPAP, PVR, PAWP, CO, cardiac index, and RAP in enrolled patients with POPH and the changes in overall mPAP, PAWP, CO, cardiac index and RAP in patients with moderate and severe POPH. For the index changes with obvious heterogeneity and ≥3 studies (overall changes in mPAP, PVR, PAWP and CO, cardiac index and RAP in all patients with POPH, and overall changes in mPAP and CO in patients with moderate and severe POPH), the sources of heterogeneity were discussed according to the following categorical variables: age, sample size, proportion of women and drug type, and no obvious sources of heterogeneity (Supplementary Tables S2, S3 for detailed data). A meta-regression analysis of the change in overall mPAP and PVR in all POPH patients and in patients with moderate and severe POPH using the continuous variables baseline mPAP and PVR was performed to explore the sources of heterogeneity. The results showed that in all POPH patients, baseline mPAP was negatively correlated with the change in PVR (*β* = −10.91558, *z* = −2.00480, *p* = 0.04498; Supplementary Table S4); in the meta-regression analysis of patients with moderate and severe POPH, the baseline PVR was positively correlated with the change in PVR (*β* = 0.00331, *z* = 3.29822, *p* = 0.00097; Supplementary Table S5), and no statistical significance was found in other regression analyses.

### 3.9 Publication bias detection

Funnel plots were made for the outcome indicators (overall changes in mPAP, PVR, PAWP, CO, cardiac index, RAP and 6MWD in all patients with POPH and overall changes in mPAP, PVR and CO in patients with moderate and severe POPH) with ≥10 studies to test publication bias. The results showed that there was no publication bias in the remaining index studies, except for an asymmetric scatter distribution corresponding to the change in overall PVR in all POPH patients, which was publication biased (t = −2.45; *p* = 0.0222; Supplementary Figure S3).

## 4 Discussion

At present, there are no clear guidelines for the specific treatment of POPH. Clinical practice is guided by PAH guidelines and expert opinions to carry out multidisciplinary treatment for patients with POPH. Due to the poor prognosis of POPH patients, few RCTs have been performed for POPH. In this meta-analysis, only one study was a randomized trial of macitentan. This meta-analysis mainly comes from retrospective studies and prospective observational studies.

### 4.1 PAH-specific treatment can significantly improve pulmonary hemodynamics in patients with POPH

Ideally, the optimal regimen of PAH-targeted drugs for POPH should reduce pulmonary artery pressure and PVR without obvious damage to liver function and improve right heart function and symptoms. In this meta-analysis, we first counted the effects of PAH treatment on mPAP, PVR, PAWP and TPG in all patients with POPH: mPAP decreased by 9.11 mmHg, PVR decreased by 239.33 dyn·s·cm^−5^, TPG decreased by 13.81 mmHg and PAWP increased by 1.36 mmHg, which were statistically significant. In current clinical practice, mPAP is used to stratify the severity of patients with POPH. If mPAP ≥35 mmHg, PAH therapy should be started for POPH. mPAP and PVR were used to assess the hemodynamics of POPH and the risk of mortality after LT. It has been reported that the posttransplant mortality in patients with moderate and severe POPH was 50%–100% (Krowka et al., 2000). In terms of treatment, the 2016 international guidelines for LT practice (Krowka et al., 2016) stated that if PAH targeted therapy leads to mPAP <35 mmHg and PVR <400 dyn·s·cm^−5^, MELD exception can be considered; if treated POPH fails to bring mPAP down to <35 mmHg, but with normal PVR (<240 dyn·s·cm^−5^) and RV function, the MELD exception can also be considered. Regarding PAWP, Swanson et al. (2008) found in their analysis that PAWP <10 mmHg was associated with death after transplantation; therefore, lowering PAWP by PAH treatment is beneficial for patients. TPG is calculated as the mean pulmonary artery pressure minus the left atrial pressure; if the TPG is high enough, it will lead to right ventricular pressure overload. Under normal circumstances, the right ventricle is a thin-walled chamber responsible for volume transmission. In the acute phase, the right ventricle is poorly adapted to the increased pressure load, which is more likely to cause right ventricular dysfunction. Overall, the above indicators improved in a “statistically significant” way (*p* < 0.05), indicating that PAH-specific treatment can significantly improve pulmonary hemodynamics in patients with POPH.

### 4.2 PAH-specific treatment significantly improved cardiac blood flow and cardiac function in patients with POPH

The meta-analysis found a 1.71 L/min increase in CO, a 0.87 L/(min·m^2^) increase in cardiac index, a 1.22 mmHg decrease in RAP, a 5.16% increase in SvO2, and a 43.41 m increase in 6MWD. The amount of blood delivered to the heart during liver graft reperfusion increased significantly, which may cause right heart failure in the already stiff and poorly compliant right ventricle (De Wolf et al., 1993; Ramsay et al., 1997; Martínez-Palli et al., 2005). The increase in CO and cardiac index after PAH treatment before LT is relatively beneficial to patients. RAP is also an indicator of right ventricular function. Recent registry data from trials evaluating early and long-term PAH disease management have shown that mean RAP is a predictor of survival (Krowka et al., 2012). Usually, patients with POPH often suffer from fatigue and insufficient exercise tolerance, which are thought to be related to insufficient cardiac output and tissue perfusion. After PAH-specific treatment, hemodynamics such as CO, cardiac index, RAP and SvO2 improved, the 6MWD increased, and the proportion of patients with NYHA and WHO FC III/IV decreased, indicating that PAH-specific treatment can significantly improve the hemodynamics and cardiac function of patients with POPH.

In the meta-analysis, we also compared the phased changes in mPAP, PVR and 6MWD.Interestingly, mPAP improved significantly after 6 months of PAH treatment, but its improvement at 1 year was not statistically significant. The reasons are as follows: 1) Hemnes and Robbins (2009) only followed 5 patients at 1 year, which was a small sample size; only 2 of these 5 patients received sildenafil 50 mg tid po, and the remaining patients received 20–25 mg, which is a low dose. Of note, although there was no statistically significant difference in hemodynamics at the first year, the mPAP decreased or remained unchanged in 4 of 5 patients, and the PVR decreased in 3 of 5 patients. 2) Reichenberger et al. (2006) followed 12 patients with RHC after 1 year of sildenafil treatment, five of whom had received prostacyclin drugs for several months before sildenafil application. 3) Hoeper et al. (2005) found that although the change in mPAP was not statistically significant after 1 year of bosentan treatment, the follow-up value decreased by 10% compared with the baseline. Therefore, although there was no “statistically” improvement in mPAP after 1 year of PAH treatment, the overall trend was improved. Next, although the 6MWD improved after 3 months of PAH treatment, the difference was not statistically significant. There was a statistical improvement after 6 months and 1 year of application. The lack of improvement in the 6MWD is related to the fact that the 6MWD may not accurately reflect the cardiopulmonary limitations of patients with severe comorbidities. In addition, the short treatment time, ascites, indication, sarcopenia, anemia and encephalopathy and other factors (Fisher et al., 2015) also make it difficult to confirm the improvement of 6MWD in some patients with POPH.

### 4.3 PAH drugs have more positive effects on cardiopulmonary hemodynamics and cardiac function in patients with moderate and severe POPH

In this meta-analysis, there were 18 studies on the treatment of moderate and severe POPH, comparing them with the changes in all POPH, and more positive changes in hemodynamic and functional status were found in patients with moderate and severe POPH treated with PAH-specific therapy. According to this result, early and appropriate PAH treatment was recommended for patients with moderate and severe POPH, considering its severity and poor prognosis. PAH treatment can reduce mPAP to <35 mmHg, allowing such patients to qualify for the MELD exception score earlier; thus, registering the waiting list for LT will be beneficial for obtaining good survival rates after LT.

### 4.4 Drug discontinution after PAH treatment

In this meta-analysis, more than half of the patients were able to stop PAH therapy after PAH therapy combined with LT. Although some patients still needed to continue treatment, they were able to maintain a good quality of life and functional status. This suggests that PAH treatment is very meaningful for patients with POPH. Khaderi et al. (2014) identified a patient with recurrence of POPH after LT, who was also the only patient with recurrent cirrhosis and portal hypertension after LT. This patient had pulmonary arterial hypertension that resolved after transplantation but recurred at the time of relapse of portal hypertension, suggesting an unexplained susceptibility to POPH.

### 4.5 Some adverse drug reactions may occur after PAH treatment

Krowka et al. (1999) found that patients treated with epoprostenol might experience different levels of facial flushing, jaw pain, dyspareunia and calf discomfort. One patient had a lower platelet count after medication. Sussman et al. (2006) reported facial flushing, nausea, anorexia and diarrhea during the application of epoprostenol and postoperative bleeding in 2 patients. This may be related to the anticoagulant effect of epoprostenol. In the study of Ashfaq et al. (2007), one patient discontinued epoprostenol due to intolerance. Hoeper et al. (2007) found that all patients tolerated inhaled iloprost well without side effects except for mild flushing, headache and cough. Several studies have reported that patients treated with ERA are prone to liver enzyme elevation, which usually recovers after dose reduction or discontinuation. Preston et al. (2020) found an asymptomatic elevation of AST (275 IU/L, more than five times the ULN) in one patient, which led to the discontinuation of ambrisentan, and the liver enzyme improved without sequelae. Hoeper et al. (2007) reported that liver aminotransferase increased to more than three times the upper limit in one patient after administration of bosentan and returned to normal after halving the dose. Sitbon et al. (2019) also reported that liver aminotransferase increased to three times or more in one patient after the application of macitentan. Therefore, in patients with POPH, ERA drugs should be used with caution if liver enzymes are elevated. Even if a patient has normal liver function, liver function needs to be monitored regularly after the application of ERA. Three studies (Cartin-Ceba et al., 2011; Halank et al., 2011; Preston et al., 2020) reported that patients developed edema after the application of Ambrisentan, which disappeared after discontinuation of the drug. Legros et al. (2017) reported a patient with moderate cytolysis after bosentan application. Fisher et al. (2015) reported that some patients experienced dyspepsia, loose stool, back pain and myalgia after PDE5 inhibitors, but the incidence was less than 10% and was generally well tolerated.

### 4.6 Heterogeneity analysis of statistical results

This meta-analysis shows that PAH treatment can improve cardiopulmonary hemodynamics and cardiac function, but there is significant heterogeneity in some statistical results (*I* ^*2*^ > 50%). We conducted a subgroup analysis but did not find a source of significant heterogeneity. This may be related to the following factors: the included studies were mainly retrospective; different studies were conducted in various periods, and the treatment regimens and follow-up times were inconsistent. Subsequently, we performed a meta-regression analysis to explore the source of heterogeneity. The results showed that in all POPH patients, the baseline mPAP was negatively correlated with the change in PVR. However, in patients with moderate and severe POPH, the baseline PVR was positively correlated with the change in PVR, while no statistical significance was found in the other regression analyses, suggesting that PVR may be an indicator affected by multiple factors, and the factors affecting changes in cardiopulmonary outcome indicators may be different for patients with different degrees of POPH, which needs to be further explored in our future studies.

### 4.7 Limitations of the meta-analysis

There were some limitations in this meta-analysis: 1) Only one of the 24 included studies was an RCT. Patients with POPH are usually excluded from prospective studies of pulmonary hypertension because of their frequent concomitant liver disease. Thus, compared with randomized controlled trials, the quality of the included studies is poor, since they mainly include observational cohort studies and retrospective case studies. 2) Most studies had relatively small samples. 3) Different studies were conducted at different periods, with different treatment schemes and inconsistent follow-up times; for this, we performed statistical analysis on some studies that had the same follow-up time. 4) Due to the lack of data regarding the severity of liver diseases (e.g., MELD score, Child–Pugh grade, *etc.*), in some studies, it was impossible to determine whether the efficacy of drugs used by patients was affected by their liver diseases. 5) We made a funnel chart for the publication bias test, and the results showed that the distribution of scattered points corresponding to the study of PVR changes was asymmetric in all POPH patients. Therefore, the results of this meta-analysis should be interpreted with caution.

## 5 Conclusion

In conclusion, PAH-specific treatment in POPH can significantly improve cardiopulmonary hemodynamics and cardiac function. As multiple drug regimens were used in clinical studies and the duration of treatment varied between studies, it is difficult to propose a specific PAH drug or drug combinations, as well as dosing regimen or duration of treatment. Considering the poor prognosis of untreated POPH patients, it is unlikely that placebo control will be used in future studies. More prospective studies or larger multicenter studies in the POPH population should be performed to confirm the current findings and adequately control for important confounding factors to expand our understanding of the effectiveness, safety, cost and optimal timing of PAH treatment in POPH patients and assist in formulating guidelines in the future. In addition, better drug regimens and treatment timing should be selected according to the clinical characteristics of patients.

## Data Availability

The original contributions presented in the study are included in the article/Supplementary Material, further inquiries can be directed to the corresponding author.

## Abbreviations

6MWD, 6-minutes walking distance; 95%*CI*, 95%confidence interval; CO, cardiac output; ERA, endothelin receptor antagonist; LT, liver transplantation; MELD, model for end-stage liver disease; mPAP, mean pulmonary artery pressure; NYHA, New York heart association; PAH, pulmonary arterial hypertension; PAWP, pulmonary arterial wedge pressure; PDE-5, phosphodiesterase type 5; POPH, portal pulmonary hypertension; PVR, pulmonary vascular resistance; RAP, right atrial pressure; RCT, randomized controlled trial; RHC, right heart catheterization; RV, right ventricle; SvO2, mixed venous oxygen saturation; TPG, transpulmonary gradient; WHO FC, WHO functional classification.
