# Supplementary material for: Efficacy evaluation of pulmonary hypertension therapy in patients with portal pulmonary hypertension: A systematic review and meta-analysis
Source: Front Pharmacol. 2022 Nov 16;13:991568. doi: 10.3389/fphar.2022.991568 (PMC9709250; doi:10.3389/fphar.2022.991568)
Supplement: Supplementary file 1 [file DataSheet1.pdf]

Supplementary Material

1 Supplementary Figures and Tables

1.1 Supplementary Figures

Fig. S1. Forest plot of hemodynamics and 6MWD in all patients with POPH. (A)mPAP: (-9.11 mmHg, 95%*CI*: -10.97~-7.26, *P*<0.0001; I^2= 54%); (B)PVR: (-239.33 dyn·s·cm-5, 95%*CI*: -272.28~-206.37; *P*<0.0001; I^2= 77%); (C)PAWP: (1.36 mmHg, 95%*CI*: 0.13~2.59; *P*= 0.0303; I^2= 74%); (D)TPG: (-13.81 mmHg, 95%*CI*: -15.95~-11.67; *P*<0.0001; I^2= 29%); (E)SvO2: (5.16%, 95%*CI*: 3.19%~ 7.14%; *P*<0.0001; I^2= 38%); (F)CO: (1.71 L/min, 95%*CI*: 1.28~2.14; *P*<0.0001; I^2= 54%); (G)Cardiac index: (0.87 L/(min·m2), 95%*CI*: 0.61~1.12; *P*<0.0001; I^2= 93%); (H)RAP: (-1.22mmHg, 95%*CI*: -2.44~-0.01; *P*= 0.0479; I^2= 62%); (I)6MWD: (43.41 m, 95%*CI*: 29.48~57.34; *P*<0.0001; I^2= 39%).

A

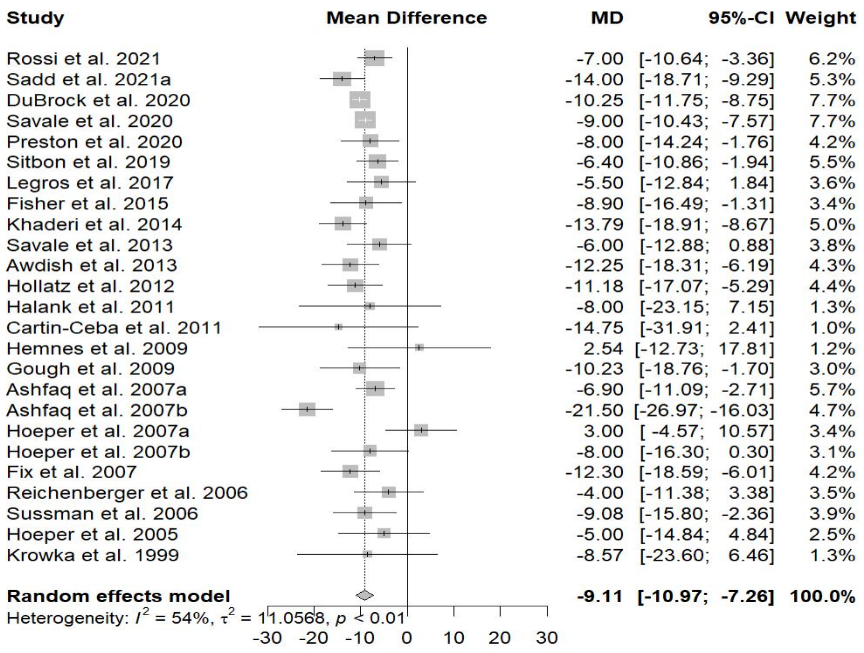

B

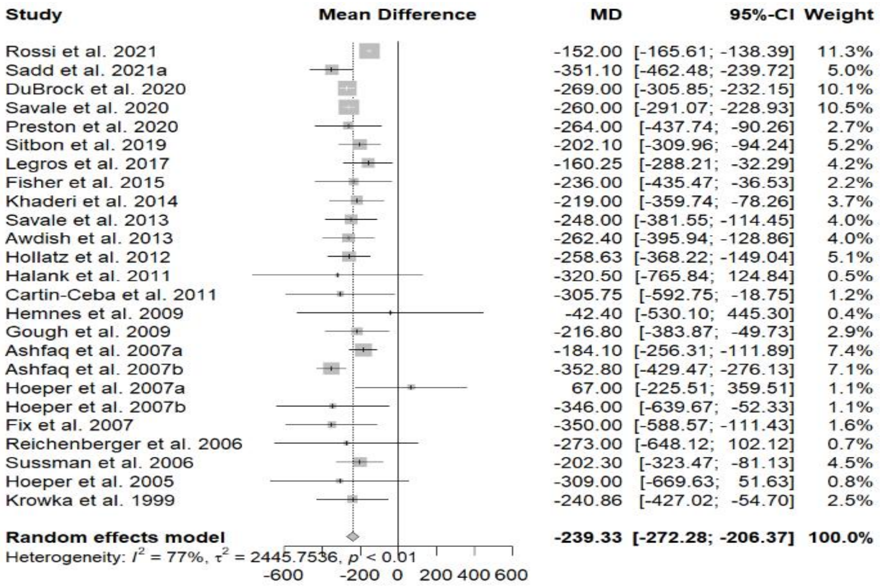
Study
Mean Difference
MD
95%-CI
Weight
Rossi et al. 2021 -152.00 [-165.61; -138.39] 11.3%
Sadd et al. 2021a -351.10 [-462.48; -239.72] 5.0%
DuBrock et al. 2020 -269.00 [-305.85; -232.15] 10.1%
Savale et al. 2020 -260.00 [-291.07; -228.93] 10.5%
Preston et al. 2020 -264.00 [-437.74; -90.26] 2.7%
Sitbon et al. 2019 -202.10 [-309.96; -94.24] 5.2%
Legros et al. 2017 -160.25 [-288.21; -32.29] 4.2%
Fisher et al. 2015 -236.00 [-435.47; -36.53] 2.2%
Khaderi et al. 2014 -219.00 [-359.74; -78.26] 3.7%
Savale et al. 2013 -248.00 [-381.55; -114.45] 4.0%
Awdish et al. 2013 -262.40 [-395.94; -128.86] 4.0%
Hollatz et al. 2012 -258.63 [-368.22; -149.04] 5.1%
Halank et al. 2011 -320.50 [-765.84; 124.84] 0.5%
Cartin-Ceba et al. 2011 -305.75 [-592.75; -18.75] 1.2%
Hemnes et al. 2009 -42.40 [-530.10; 445.30] 0.4%
Gough et al. 2009 -216.80 [-383.87; -49.73] 2.9%
Ashfaq et al. 2007a -184.10 [-256.31; -111.89] 7.4%
Ashfaq et al. 2007b -352.80 [-429.47; -276.13] 7.1%
Hoeper et al. 2007a 67.00 [-225.51; 359.51] 1.1%
Hoeper et al. 2007b -346.00 [-639.67; -52.33] 1.1%
Fix et al. 2007 -350.00 [-588.57; -111.43] 1.6%
Reichenberger et al. 2006 -273.00 [-648.12; 102.12] 0.7%
Sussman et al. 2006 -202.30 [-323.47; -81.13] 4.5%
Hoeper et al. 2005 -309.00 [-669.63; 51.63] 0.8%
Krowka et al. 1999 -240.86 [-427.02; -54.70] 2.5%
Random effects model -239.33 [-272.28; -206.37] 100.0%
Heterogeneity: I^2 = 77%, τ^2 = 2445.7536, p < 0.01
-600 -200 0 200 400 600

C

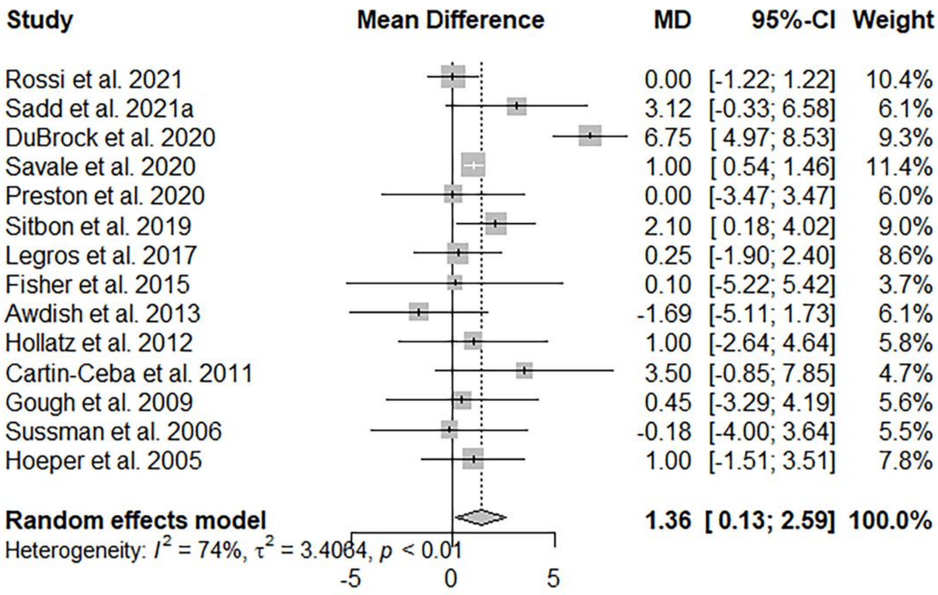
Study
Mean Difference
MD
95%-CI
Weight
Rossi et al. 2021 0.00 [-1.22; 1.22] 10.4%
Sadd et al. 2021a 3.12 [-0.33; 6.58] 6.1%
DuBrock et al. 2020 6.75 [4.97; 8.53] 9.3%
Savale et al. 2020 1.00 [0.54; 1.46] 11.4%
Preston et al. 2020 0.00 [-3.47; 3.47] 6.0%
Sitbon et al. 2019 2.10 [0.18; 4.02] 9.0%
Legros et al. 2017 0.25 [-1.90; 2.40] 8.6%
Fisher et al. 2015 0.10 [-5.22; 5.42] 3.7%
Awdish et al. 2013 -1.69 [-5.11; 1.73] 6.1%
Hollatz et al. 2012 1.00 [-2.64; 4.64] 5.8%
Cartin-Ceba et al. 2011 3.50 [-0.85; 7.85] 4.7%
Gough et al. 2009 0.45 [-3.29; 4.19] 5.6%
Sussman et al. 2006 -0.18 [-4.00; 3.64] 5.5%
Hoeper et al. 2005 1.00 [-1.51; 3.51] 7.8%
Random effects model 1.36 [0.13; 2.59] 100.0%
Heterogeneity: I^2 = 74%, τ^2 = 3.4064, p < 0.01
-5 0 5

D

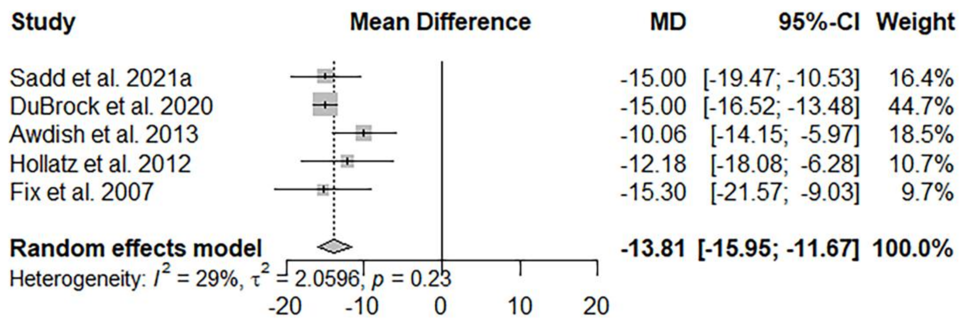
Study
Mean Difference
MD
95%-CI
Weight
Sadd et al. 2021a
-15.00 [-19.47; -10.53] 16.4%
DuBrock et al. 2020
-15.00 [-16.52; -13.48] 44.7%
Awdish et al. 2013
-10.06 [-14.15; -5.97] 18.5%
Hollatz et al. 2012
-12.18 [-18.08; -6.28] 10.7%
Fix et al. 2007
-15.30 [-21.57; -9.03] 9.7%
Random effects model
-13.81 [-15.95; -11.67] 100.0%
Heterogeneity: I^2 = 29%, τ^2 = 2.0596, p = 0.23
-20 -10 0 10 20

E

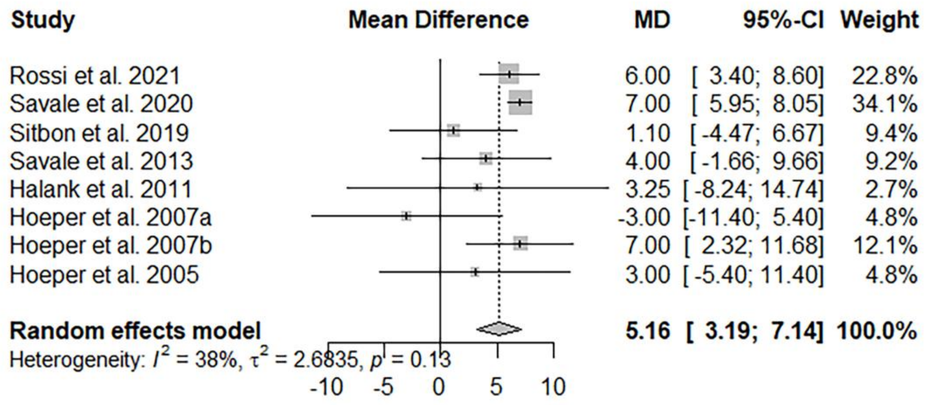
Study
Mean Difference
MD
95%-CI
Weight
Rossi et al. 2021
6.00 [3.40; 8.60] 22.8%
Savale et al. 2020
7.00 [5.95; 8.05] 34.1%
Sitbon et al. 2019
1.10 [-4.47; 6.67] 9.4%
Savale et al. 2013
4.00 [-1.66; 9.66] 9.2%
Halank et al. 2011
3.25 [-8.24; 14.74] 2.7%
Hoeper et al. 2007a
-3.00 [-11.40; 5.40] 4.8%
Hoeper et al. 2007b
7.00 [2.32; 11.68] 12.1%
Hoeper et al. 2005
3.00 [-5.40; 11.40] 4.8%
Random effects model
5.16 [3.19; 7.14] 100.0%
Heterogeneity: I^2 = 38%, τ^2 = 2.6835, p = 0.13
-10 -5 0 5 10

F

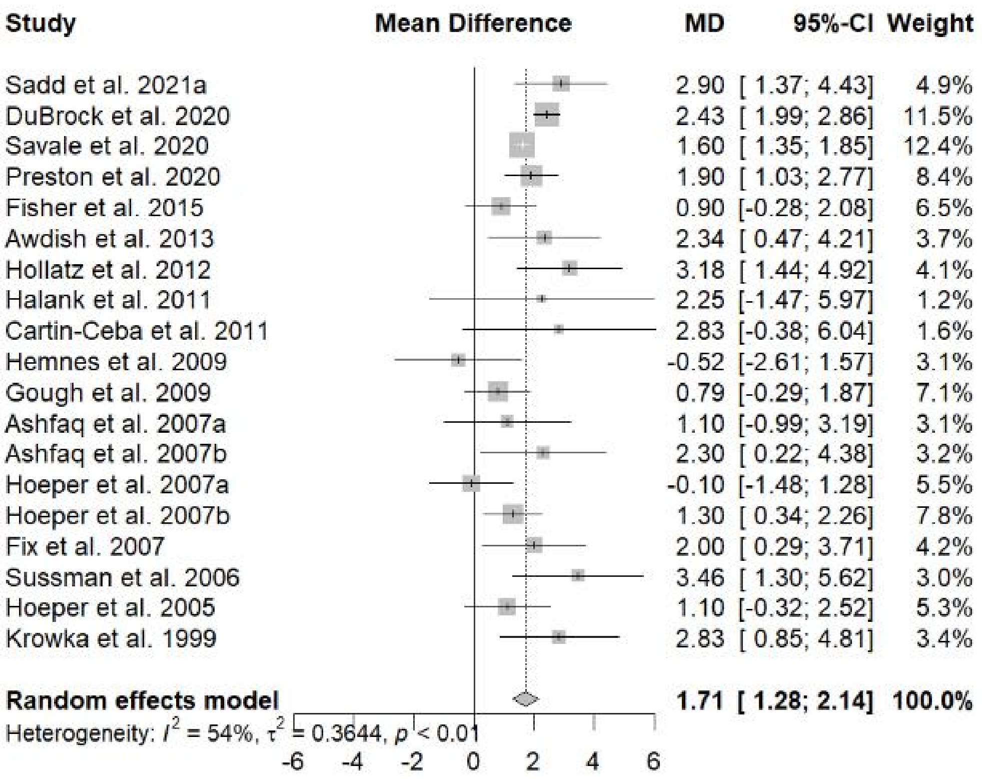
Study
Mean Difference
MD
95%-CI
Weight
Sadd et al. 2021a 2.90 [1.37; 4.43] 4.9%
DuBrock et al. 2020 2.43 [1.99; 2.86] 11.5%
Savale et al. 2020 1.60 [1.35; 1.85] 12.4%
Preston et al. 2020 1.90 [1.03; 2.77] 8.4%
Fisher et al. 2015 0.90 [-0.28; 2.08] 6.5%
Awdish et al. 2013 2.34 [0.47; 4.21] 3.7%
Hollatz et al. 2012 3.18 [1.44; 4.92] 4.1%
Halank et al. 2011 2.25 [-1.47; 5.97] 1.2%
Cartin-Ceba et al. 2011 2.83 [-0.38; 6.04] 1.6%
Hemnes et al. 2009 -0.52 [-2.61; 1.57] 3.1%
Gough et al. 2009 0.79 [-0.29; 1.87] 7.1%
Ashfaq et al. 2007a 1.10 [-0.99; 3.19] 3.1%
Ashfaq et al. 2007b 2.30 [0.22; 4.38] 3.2%
Hoeper et al. 2007a -0.10 [-1.48; 1.28] 5.5%
Hoeper et al. 2007b 1.30 [0.34; 2.26] 7.8%
Fix et al. 2007 2.00 [0.29; 3.71] 4.2%
Sussman et al. 2006 3.46 [1.30; 5.62] 3.0%
Hoeper et al. 2005 1.10 [-0.32; 2.52] 5.3%
Krowka et al. 1999 2.83 [0.85; 4.81] 3.4%
Random effects model 1.71 [1.28; 2.14] 100.0%
Heterogeneity: I^2 = 54%, τ^2 = 0.3644, p < 0.01
-6 -4 -2 0 2 4 6

G

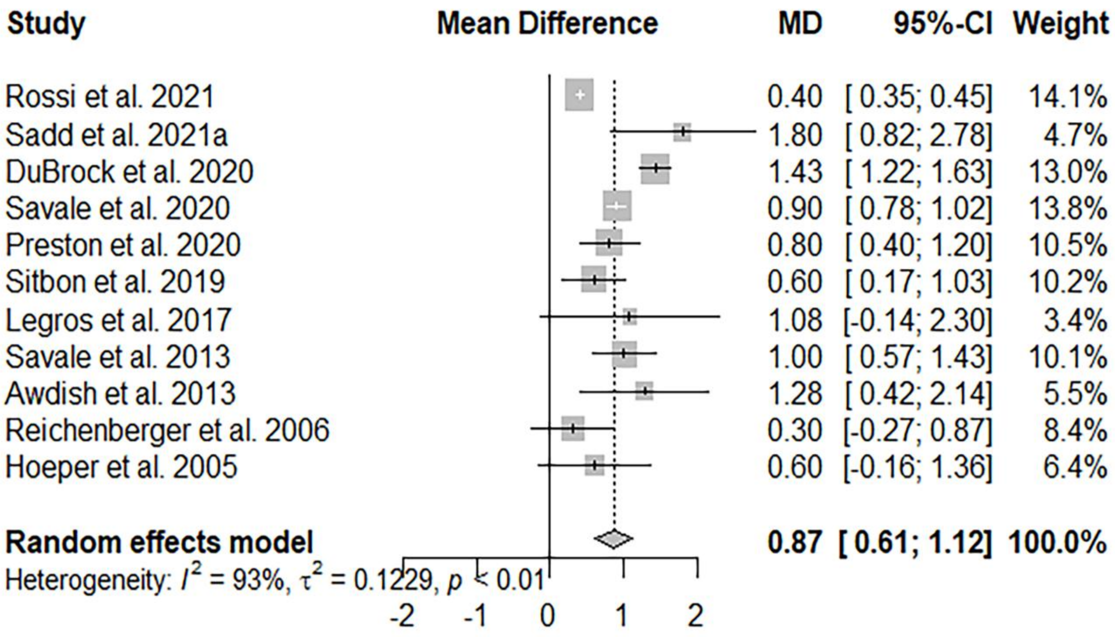
Study
Mean Difference
MD
95%-CI
Weight
Rossi et al. 2021 0.40 [0.35; 0.45] 14.1%
Sadd et al. 2021a 1.80 [0.82; 2.78] 4.7%
DuBrock et al. 2020 1.43 [1.22; 1.63] 13.0%
Savale et al. 2020 0.90 [0.78; 1.02] 13.8%
Preston et al. 2020 0.80 [0.40; 1.20] 10.5%
Sitbon et al. 2019 0.60 [0.17; 1.03] 10.2%
Legros et al. 2017 1.08 [-0.14; 2.30] 3.4%
Savale et al. 2013 1.00 [0.57; 1.43] 10.1%
Awdish et al. 2013 1.28 [0.42; 2.14] 5.5%
Reichenberger et al. 2006 0.30 [-0.27; 0.87] 8.4%
Hoeper et al. 2005 0.60 [-0.16; 1.36] 6.4%
Random effects model 0.87 [0.61; 1.12] 100.0%
Heterogeneity: I^2 = 93%, τ^2 = 0.1229, p < 0.01
-2 -1 0 1 2

H

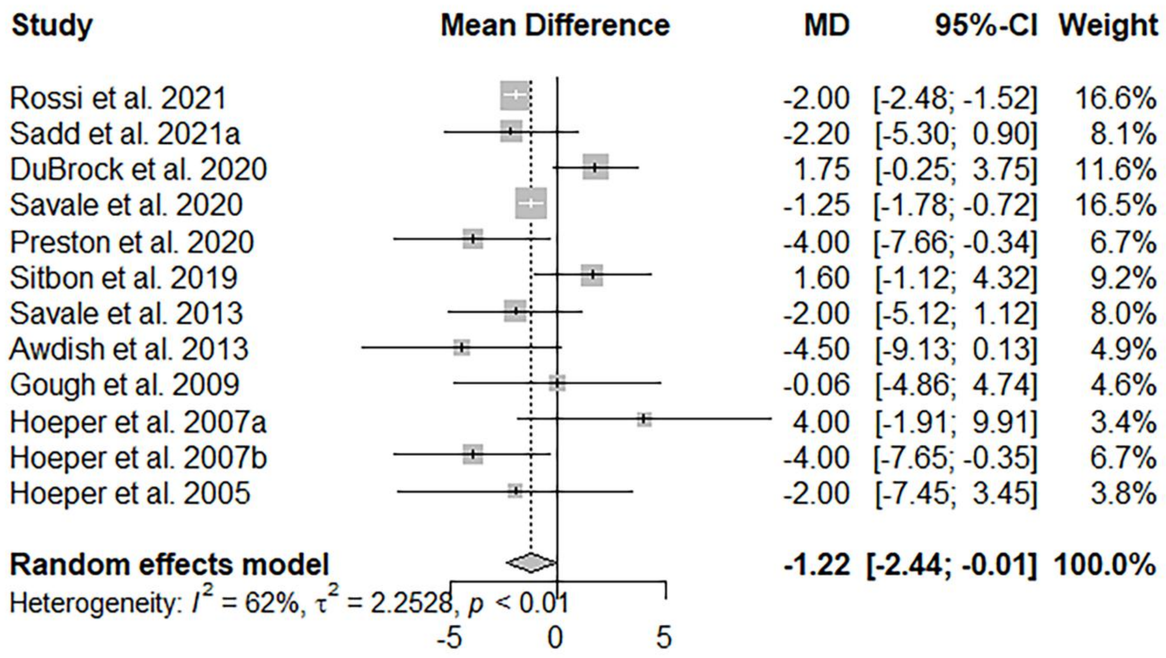
Study
Mean Difference
MD
95%-CI
Weight
Rossi et al. 2021 -2.00 [-2.48; -1.52] 16.6%
Sadd et al. 2021a -2.20 [-5.30; 0.90] 8.1%
DuBrock et al. 2020 1.75 [-0.25; 3.75] 11.6%
Savale et al. 2020 -1.25 [-1.78; -0.72] 16.5%
Preston et al. 2020 -4.00 [-7.66; -0.34] 6.7%
Sitbon et al. 2019 1.60 [-1.12; 4.32] 9.2%
Savale et al. 2013 -2.00 [-5.12; 1.12] 8.0%
Awdish et al. 2013 -4.50 [-9.13; 0.13] 4.9%
Gough et al. 2009 -0.06 [-4.86; 4.74] 4.6%
Hoeper et al. 2007a 4.00 [-1.91; 9.91] 3.4%
Hoeper et al. 2007b -4.00 [-7.65; -0.35] 6.7%
Hoeper et al. 2005 -2.00 [-7.45; 3.45] 3.8%
Random effects model -1.22 [-2.44; -0.01] 100.0%
Heterogeneity: I^2 = 62%, τ^2 = 2.2528, p < 0.01
-5
0
5

I

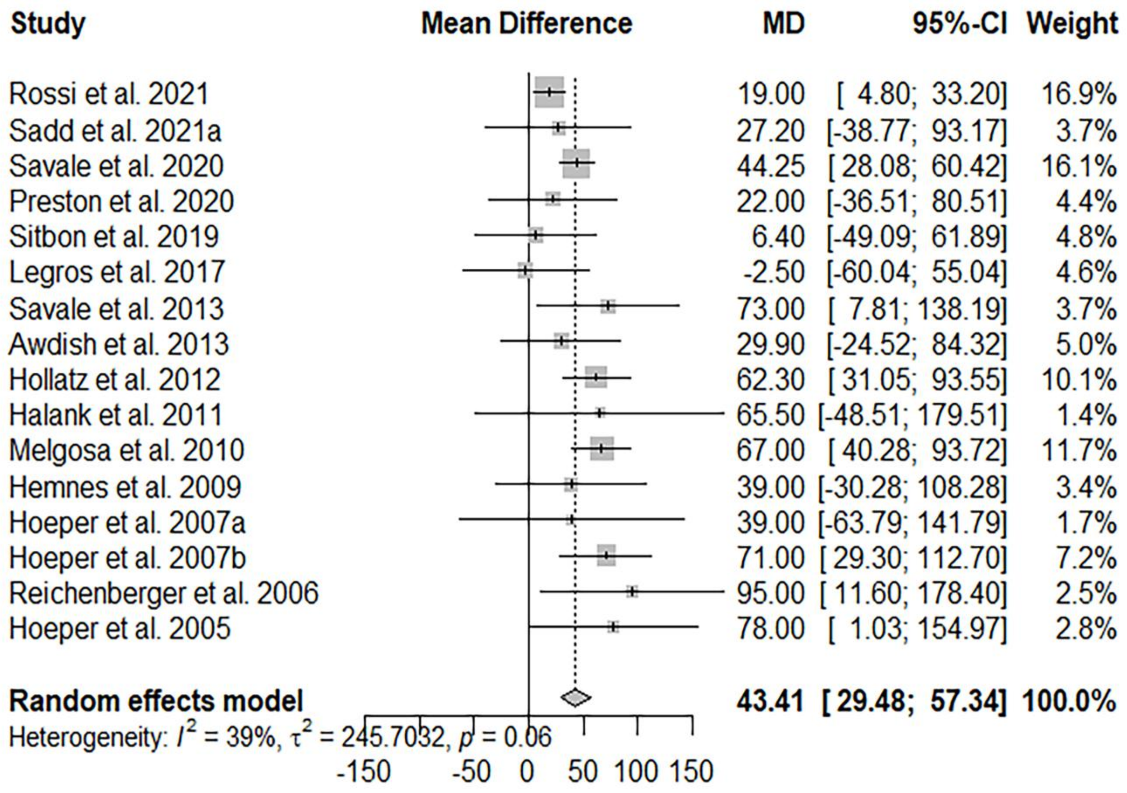
Study
Mean Difference
MD
95%-CI
Weight
Rossi et al. 2021 19.00 [4.80; 33.20] 16.9%
Sadd et al. 2021a 27.20 [-38.77; 93.17] 3.7%
Savale et al. 2020 44.25 [28.08; 60.42] 16.1%
Preston et al. 2020 22.00 [-36.51; 80.51] 4.4%
Sitbon et al. 2019 6.40 [-49.09; 61.89] 4.8%
Legros et al. 2017 -2.50 [-60.04; 55.04] 4.6%
Savale et al. 2013 73.00 [7.81; 138.19] 3.7%
Awdish et al. 2013 29.90 [-24.52; 84.32] 5.0%
Hollatz et al. 2012 62.30 [31.05; 93.55] 10.1%
Halank et al. 2011 65.50 [-48.51; 179.51] 1.4%
Melgosa et al. 2010 67.00 [40.28; 93.72] 11.7%
Hemnes et al. 2009 39.00 [-30.28; 108.28] 3.4%
Hoeper et al. 2007a 39.00 [-63.79; 141.79] 1.7%
Hoeper et al. 2007b 71.00 [29.30; 112.70] 7.2%
Reichenberger et al. 2006 95.00 [11.60; 178.40] 2.5%
Hoeper et al. 2005 78.00 [1.03; 154.97] 2.8%
Random effects model 43.41 [29.48; 57.34] 100.0%
Heterogeneity: I^2 = 39%, τ^2 = 245.7032, p = 0.06
-150
-50
0
50
100
150

Fig.S2. Forest plot of hemodynamics and 6MWD in patients with moderate and severe POPH. (A)SvO2 (3.36%, 95%*CI*: -1.46%~8.19%; *P*= 0.1714; I^2= 30%); (B)RAP(-0.53 mmHg, 95%*CI*: -2.83~1.76; *P*= 0.6473; I^2= 57%); (C)mPAP: (-9.63 mmHg, 95%*CI*: -12.49~-6.78; *P*<0.0001; I^2= 62%); (D)PVR: (-259.78 dyn·s·cm-5, 95%*CI*: -301.56~-218.01; *P*<0.0001; I^2= 23%); (E)PAWP: (2.45 mmHg, 95%*CI*: 0.36~4.54; *P*= 0.0217; I^2= 76%); (F)TPG: (-14.86 mmHg, 95%*CI*: -16.23~-13.50; *P*<0.0001; I^2= 0%); (G)CO: (1.76 L/min, 95%*CI*: 1.16~2.36; *P*<0.0001; I^2= 58%); (H)Cardiac index: (1.01 L/(min·m2), 95%*CI*: 0.35~1.67; *P*= 0.0027; I^2= 83%); (I)6MWD: (61.30 m, 95%*CI*: 41.38~81.21; *P*<0.0001; I^2= 0%).

A

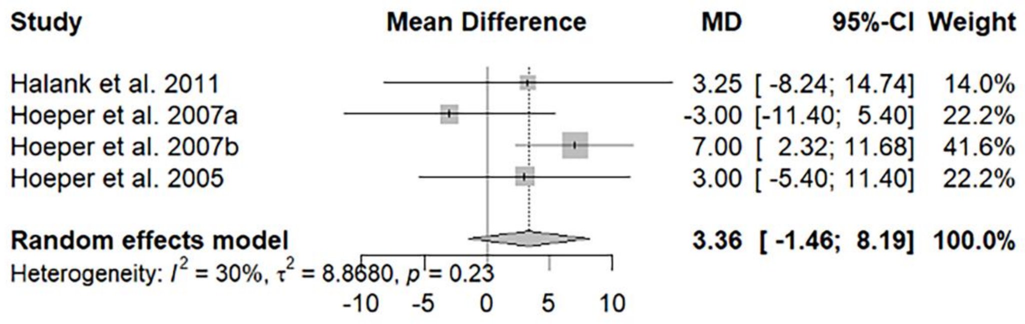

B

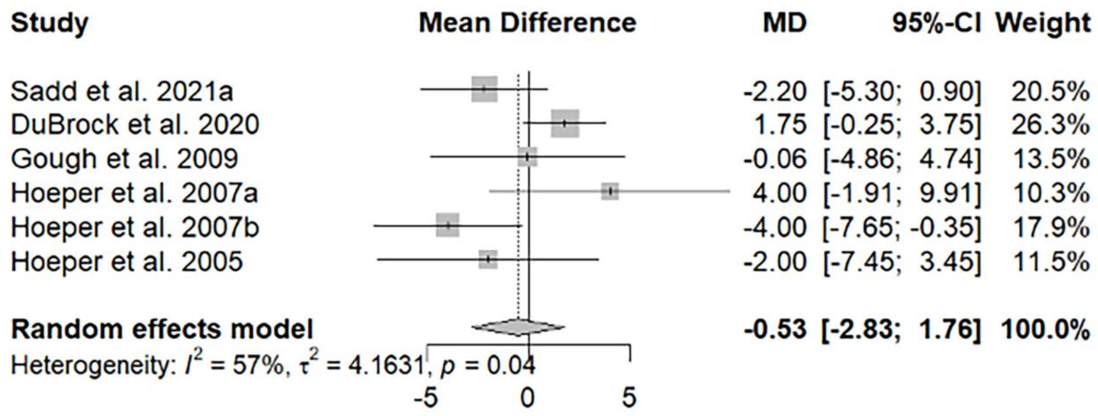

C

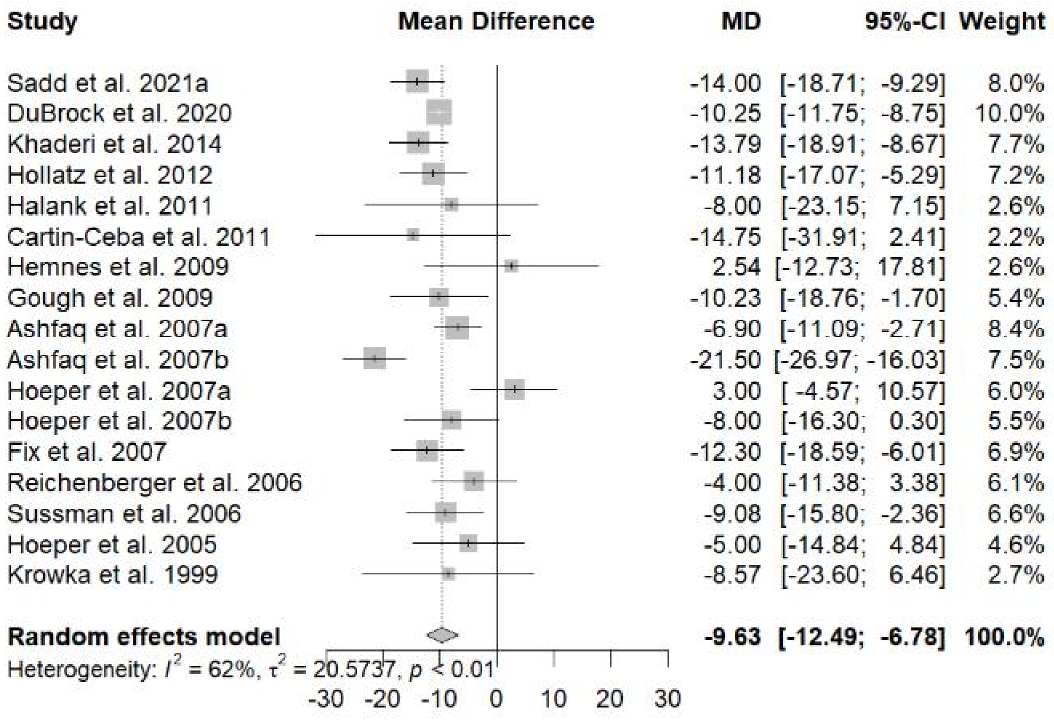
Study
Mean Difference
MD
95%-CI
Weight
Sadd et al. 2021a -14.00 [-18.71; -9.29] 8.0%
DuBrock et al. 2020 -10.25 [-11.75; -8.75] 10.0%
Khaderi et al. 2014 -13.79 [-18.91; -8.67] 7.7%
Hollatz et al. 2012 -11.18 [-17.07; -5.29] 7.2%
Halank et al. 2011 -8.00 [-23.15; 7.15] 2.6%
Cartin-Ceba et al. 2011 -14.75 [-31.91; 2.41] 2.2%
Hemnes et al. 2009 2.54 [-12.73; 17.81] 2.6%
Gough et al. 2009 -10.23 [-18.76; -1.70] 5.4%
Ashfaq et al. 2007a -6.90 [-11.09; -2.71] 8.4%
Ashfaq et al. 2007b -21.50 [-26.97; -16.03] 7.5%
Hoeper et al. 2007a 3.00 [-4.57; 10.57] 6.0%
Hoeper et al. 2007b -8.00 [-16.30; 0.30] 5.5%
Fix et al. 2007 -12.30 [-18.59; -6.01] 6.9%
Reichenberger et al. 2006 -4.00 [-11.38; 3.38] 6.1%
Sussman et al. 2006 -9.08 [-15.80; -2.36] 6.6%
Hoeper et al. 2005 -5.00 [-14.84; 4.84] 4.6%
Krowka et al. 1999 -8.57 [-23.60; 6.46] 2.7%
Random effects model -9.63 [-12.49; -6.78] 100.0%
Heterogeneity: I^2 = 62%, τ^2 = 20.5737, p < 0.01
-30 -20 -10 0 10 20 30

D

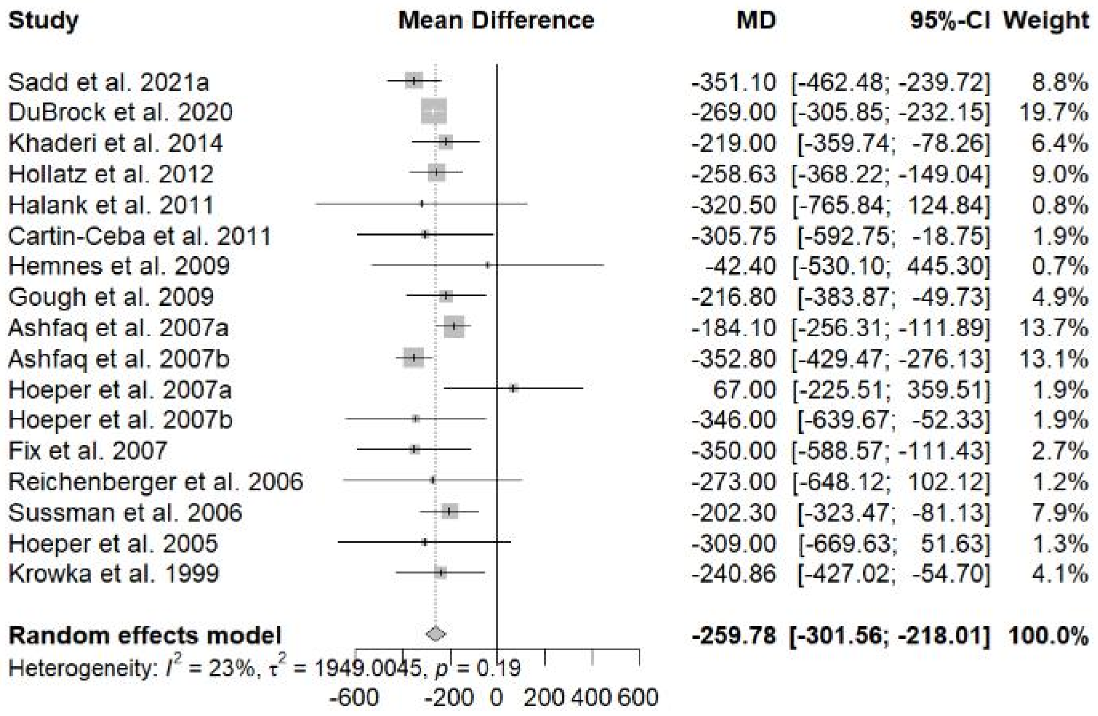
Study
Mean Difference
MD
95%-CI
Weight
Sadd et al. 2021a -351.10 [-462.48; -239.72] 8.8%
DuBrock et al. 2020 -269.00 [-305.85; -232.15] 19.7%
Khaderi et al. 2014 -219.00 [-359.74; -78.26] 6.4%
Hollatz et al. 2012 -258.63 [-368.22; -149.04] 9.0%
Halank et al. 2011 -320.50 [-765.84; 124.84] 0.8%
Cartin-Ceba et al. 2011 -305.75 [-592.75; -18.75] 1.9%
Hemnes et al. 2009 -42.40 [-530.10; 445.30] 0.7%
Gough et al. 2009 -216.80 [-383.87; -49.73] 4.9%
Ashfaq et al. 2007a -184.10 [-256.31; -111.89] 13.7%
Ashfaq et al. 2007b -352.80 [-429.47; -276.13] 13.1%
Hoeper et al. 2007a 67.00 [-225.51; 359.51] 1.9%
Hoeper et al. 2007b -346.00 [-639.67; -52.33] 1.9%
Fix et al. 2007 -350.00 [-588.57; -111.43] 2.7%
Reichenberger et al. 2006 -273.00 [-648.12; 102.12] 1.2%
Sussman et al. 2006 -202.30 [-323.47; -81.13] 7.9%
Hoeper et al. 2005 -309.00 [-669.63; 51.63] 1.3%
Krowka et al. 1999 -240.86 [-427.02; -54.70] 4.1%
Random effects model -259.78 [-301.56; -218.01] 100.0%
Heterogeneity: I^2 = 23%, τ^2 = 1949.0045, p = 0.19
-600 -200 0 200 400 600

E

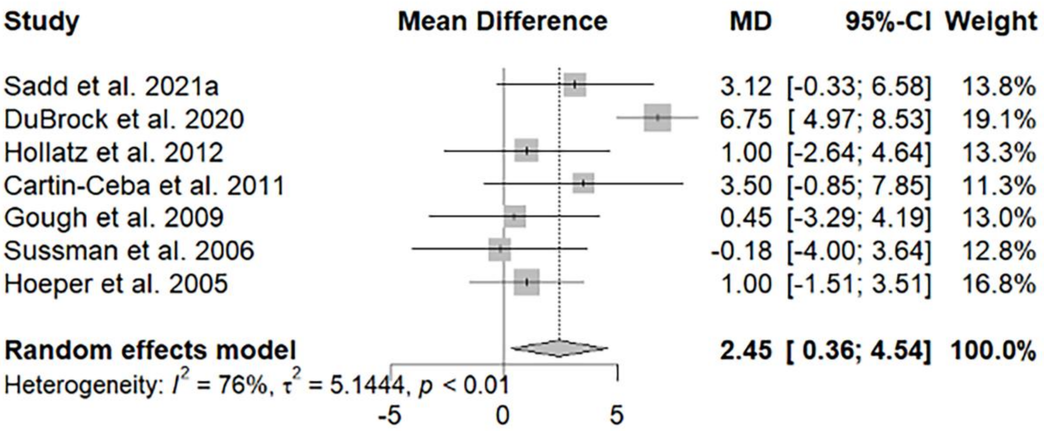
Study
Mean Difference
MD
95%-CI
Weight
Sadd et al. 2021a
3.12 [-0.33; 6.58] 13.8%
DuBrock et al. 2020
6.75 [4.97; 8.53] 19.1%
Hollatz et al. 2012
1.00 [-2.64; 4.64] 13.3%
Cartin-Ceba et al. 2011
3.50 [-0.85; 7.85] 11.3%
Gough et al. 2009
0.45 [-3.29; 4.19] 13.0%
Sussman et al. 2006
-0.18 [-4.00; 3.64] 12.8%
Hoeper et al. 2005
1.00 [-1.51; 3.51] 16.8%
Random effects model
2.45 [0.36; 4.54] 100.0%
Heterogeneity: I^2 = 76%, τ^2 = 5.1444, p < 0.01
-5
0
5

F

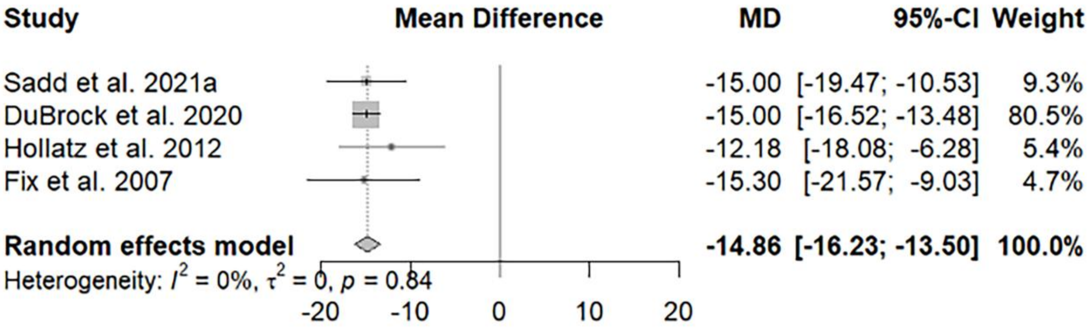
Study
Mean Difference
MD
95%-CI
Weight
Sadd et al. 2021a
-15.00 [-19.47; -10.53] 9.3%
DuBrock et al. 2020
-15.00 [-16.52; -13.48] 80.5%
Hollatz et al. 2012
-12.18 [-18.08; -6.28] 5.4%
Fix et al. 2007
-15.30 [-21.57; -9.03] 4.7%
Random effects model
-14.86 [-16.23; -13.50] 100.0%
Heterogeneity: I^2 = 0%, τ^2 = 0, p = 0.84
-20
-10
0
10
20

G

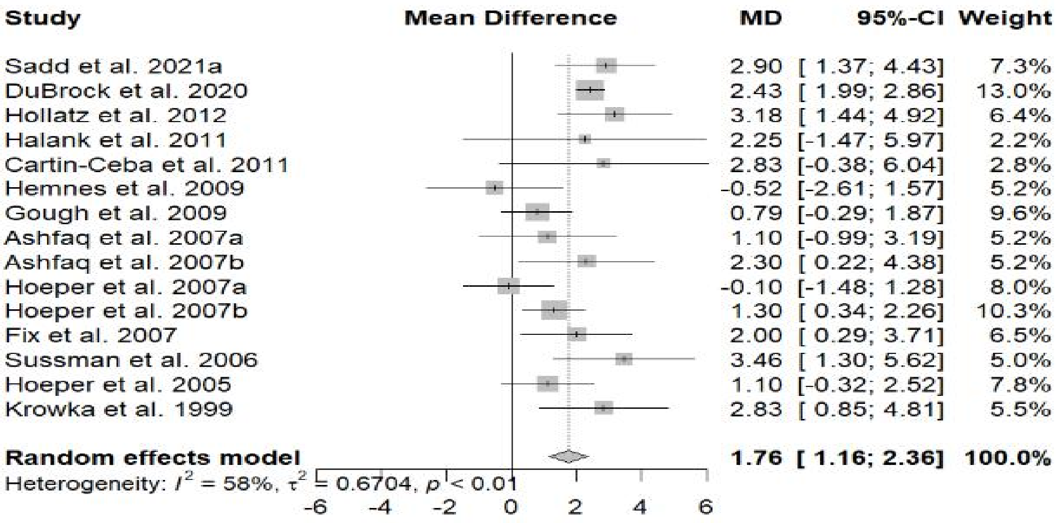
Study
Mean Difference
MD
95%-CI
Weight
Sadd et al. 2021a
2.90 [1.37; 4.43] 7.3%
DuBrock et al. 2020
2.43 [1.99; 2.86] 13.0%
Hollatz et al. 2012
3.18 [1.44; 4.92] 6.4%
Halank et al. 2011
2.25 [-1.47; 5.97] 2.2%
Cartin-Ceba et al. 2011
2.83 [-0.38; 6.04] 2.8%
Hemnes et al. 2009
-0.52 [-2.61; 1.57] 5.2%
Gough et al. 2009
0.79 [-0.29; 1.87] 9.6%
Ashfaq et al. 2007a
1.10 [-0.99; 3.19] 5.2%
Ashfaq et al. 2007b
2.30 [0.22; 4.38] 5.2%
Hoeper et al. 2007a
-0.10 [-1.48; 1.28] 8.0%
Hoeper et al. 2007b
1.30 [0.34; 2.26] 10.3%
Fix et al. 2007
2.00 [0.29; 3.71] 6.5%
Sussman et al. 2006
3.46 [1.30; 5.62] 5.0%
Hoeper et al. 2005
1.10 [-0.32; 2.52] 7.8%
Krowka et al. 1999
2.83 [0.85; 4.81] 5.5%
Random effects model
1.76 [1.16; 2.36] 100.0%
Heterogeneity: I^2 = 58%, τ^2 = 0.6704, p < 0.01
-6
-4
-2
0
2
4
6

H

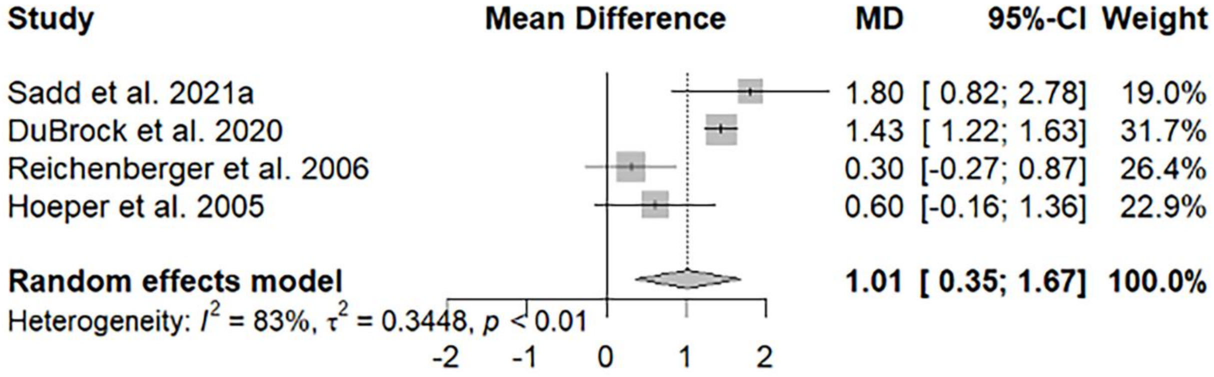
Study
Mean Difference
MD
95%-CI
Weight
Sadd et al. 2021a
1.80 [0.82; 2.78] 19.0%
DuBrock et al. 2020
1.43 [1.22; 1.63] 31.7%
Reichenberger et al. 2006
0.30 [-0.27; 0.87] 26.4%
Hoeper et al. 2005
0.60 [-0.16; 1.36] 22.9%
Random effects model
1.01 [0.35; 1.67] 100.0%
Heterogeneity: I^2 = 83%, τ^2 = 0.3448, p < 0.01
-2 -1 0 1 2

I

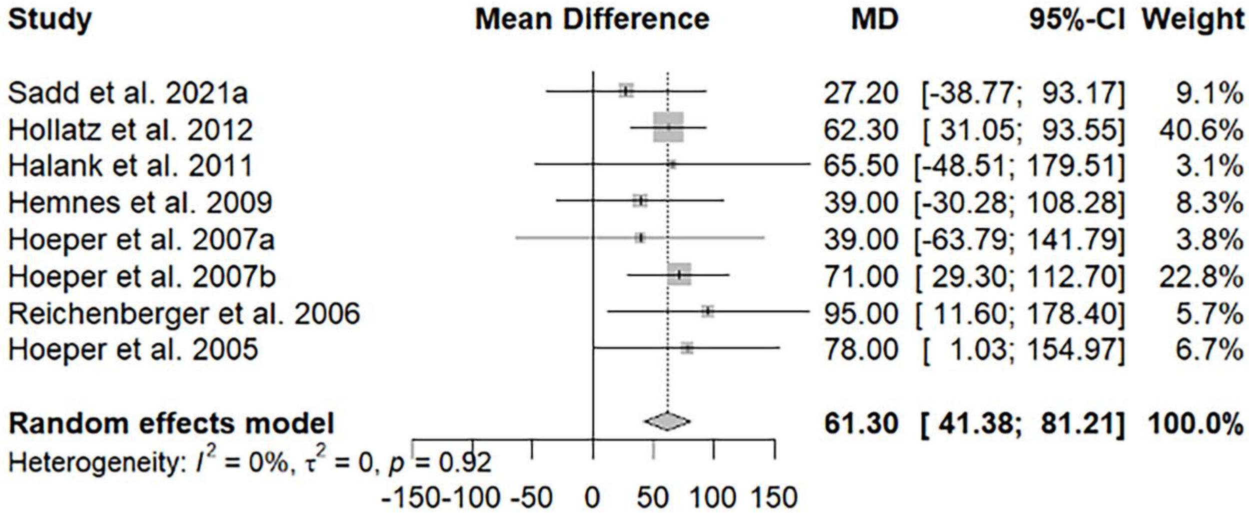
Study
Mean Difference
MD
95%-CI
Weight
Sadd et al. 2021a
27.20 [-38.77; 93.17] 9.1%
Hollatz et al. 2012
62.30 [31.05; 93.55] 40.6%
Halank et al. 2011
65.50 [-48.51; 179.51] 3.1%
Hemnes et al. 2009
39.00 [-30.28; 108.28] 8.3%
Hoeper et al. 2007a
39.00 [-63.79; 141.79] 3.8%
Hoeper et al. 2007b
71.00 [29.30; 112.70] 22.8%
Reichenberger et al. 2006
95.00 [11.60; 178.40] 5.7%
Hoeper et al. 2005
78.00 [1.03; 154.97] 6.7%
Random effects model
61.30 [41.38; 81.21] 100.0%
Heterogeneity: I^2 = 0%, τ^2 = 0, p = 0.92
-150-100 -50 0 50 100 150

Fig. S3. Funnel diagrams of studies for inclusion in the meta-analysis. (A) Overall mPAP changes in all POPH patients (t=0.62; *P*=0.5385). (B) Overall PVR changes in all POPH patients (t= -2.45; *P*=0.0222). (C) Overall PAWP changes in all POPH patients (t=0.36; *P*=0.7228). (D) Overalll CO changes in all POPH patients (t=0.08; *P*=0.9386). (E) Overall Cardiac index changes in all POPH patients (t=1.89; *P*=0.0919). (F) Overall RAP changes in all POPH patients (t=0.59; *P*=0.5671). (G) Overall 6MWD changes in all POPH patients (t=1.08; *P*=0.2988). (H) Overall mPAP changes in patients with moderate and severe POPH (t=0.62; *P*=0.5438). (I) Overall PVR changes in patients with moderate and severe POPH (t=0.54; *P*=0.5998). (J) Overall CO changes in patients with moderate and severe POPH (t= -0.84; *P*=0.4144).

Note: 6MWD, 6-minutes walking distance; CO, cardiac output; mPAP, mean pulmonary artery pressure; PAWP, Pulmonary wedge pressure; PVR, pulmonary vascular resistance; RAP, right atrial pressure; TPG, Transpulmonary gradient.

A

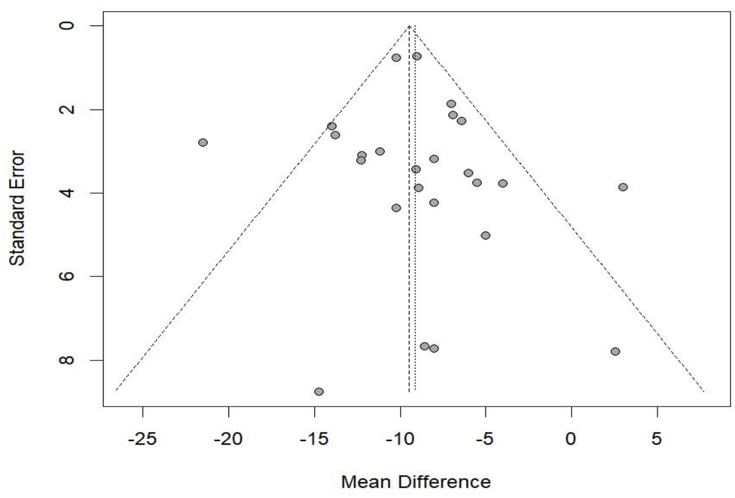

B

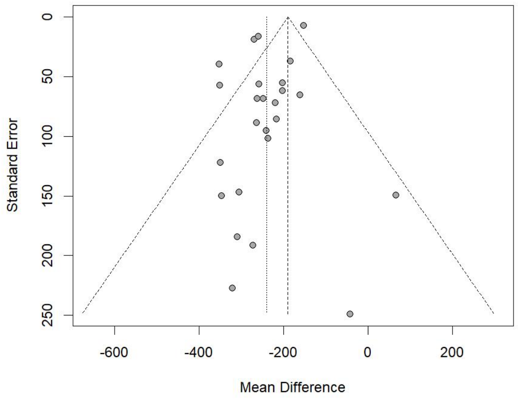
Standard Error
0
50
100
150
200
250
-600
-400
-200
0
200
Mean Difference

C

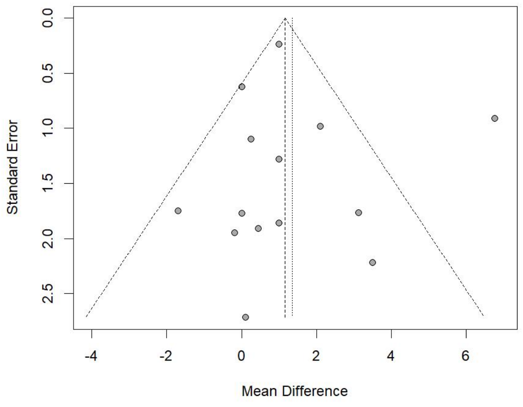
Standard Error
0.0
0.5
1.0
1.5
2.0
2.5
-4
-2
0
2
4
6
Mean Difference

D

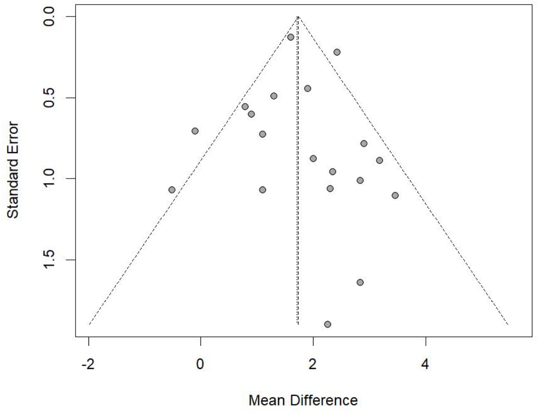
Standard Error
0.0
0.5
1.0
1.5
-2
0
2
4
Mean Difference

E

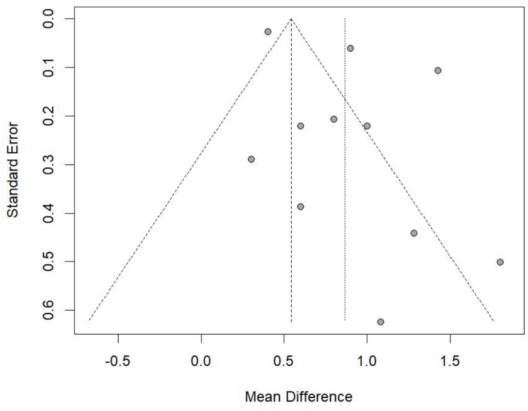
Standard Error
0.0
0.1
0.2
0.3
0.4
0.5
0.6
-0.5
0.0
0.5
1.0
1.5
Mean Difference

F

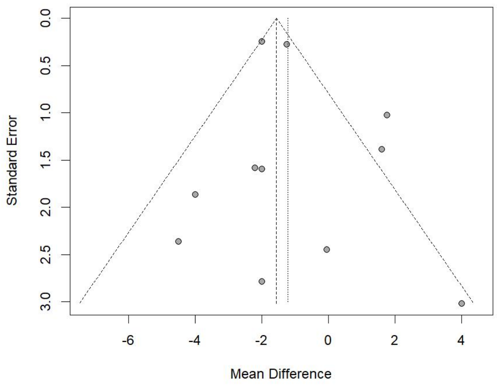
Standard Error
0.0
0.5
1.0
1.5
2.0
2.5
3.0
-6
-4
-2
0
2
4
Mean Difference

G

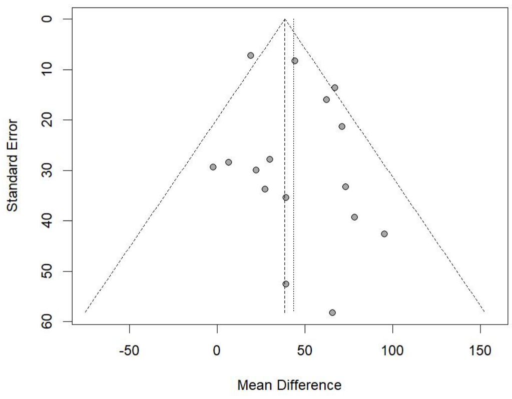
Standard Error
0
10
20
30
40
50
60
-50
0
50
100
150
Mean Difference

H

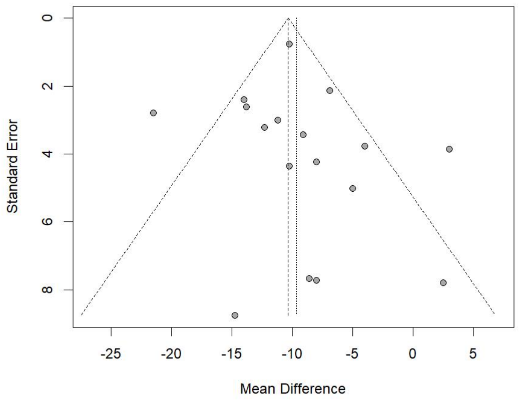
Standard Error
Mean Difference
0
2
4
6
8
-25
-20
-15
-10
-5
0
5

I

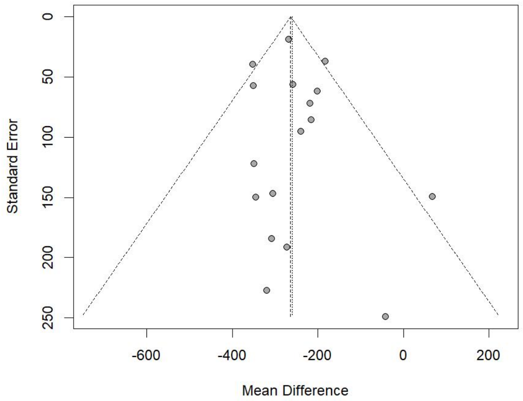
Standard Error
Mean Difference
0
50
100
150
200
250
-600
-400
-200
0
200

J

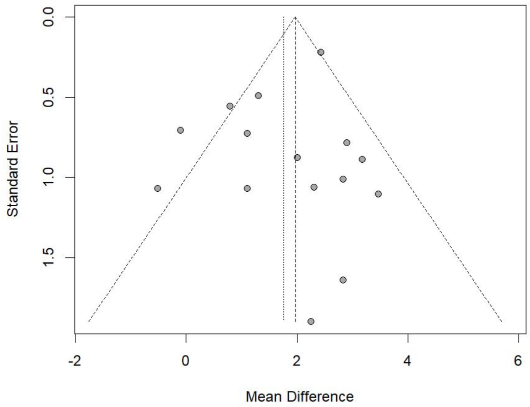
Standard Error
Mean Difference
0.0
0.5
1.0
1.5
-2
0
2
4
6

1.2 Supplementary Tables

Table S1. Quality assessment of studies for inclusion in the meta-analysis.

First author	Publication year	1. Was the study question or objective clearly stated?	2. Was the study population clearly fully described, including a case definition?	3. Were the cases consecutive?	4. Were the subjects comparable?	5. Was the intervention clearly described?	6. Were the outcome measures clearly defined, valid, reliable, and implemented consistently across all study participants?	7. Was the length of follow-up adequate?	8. Were the statistical methods well described?	9. Were the study results well described?
Rossi. R	2021	YES	YES	YES	YES	YES	YES	YES	YES	YES
Sadd. C J	2021	YES	YES	CD	YES	NO	YES	YES	YES	YES
DuBrock.H M	2020	YES	YES	CD	YES	YES	YES	YES	NO	YES
Savale .L	2020	YES	YES	YES	YES	YES	YES	YES	YES	YES

Preston.I R	2020	YES	YES	CD	YES	YES	YES	YES	YES	YES
Legros .L	2017	YES	YES	CD	YES	YES	YES	YES	YES	YES
Fisher. J H	2015	YES	YES	CD	YES	YES	YES	YES	YES	YES
Khaderi.S	2014	YES	YES	CD	YES	YES	YES	YES	NO	YES
Savale .L	2013	YES	YES	YES	YES	YES	YES	YES	YES	YES
Awdish.R L A	2013	YES	YES	CD	YES	YES	YES	YES	YES	YES
Hollatz.T J	2012	YES	YES	CD	YES	YES	YES	YES	YES	YES
Halank.M	2011	YES	YES	CD	YES	YES	YES	YES	YES	YES
Cartin-Ceba. R	2011	YES	YES	YES	YES	YES	YES	YES	YES	YES
Melgosa.M T	2010	YES	NO	YES	YES	YES	YES	YES	YES	YES
Hemnes.A R	2009	YES	YES	CD	YES	YES	YES	YES	YES	YES
Gough .M S	2009	YES	YES	YES	YES	YES	YES	YES	NO	YES

Ashfaq.M	2007	YES	YES	CD	YES	YES	YES	YES	YES	YES
Hoeper.M M	2007	YES	NO	YES	YES	YES	YES	YES	YES	YES
Fix.O K	2007	YES	YES	CD	YES	YES	YES	YES	YES	YES
Reichenberger	2006	YES	YES	CD	YES	YES	YES	YES	YES	YES
Sussman.N	2006	YES	YES	YES	YES	YES	YES	YES	YES	YES
Hoeper.M M	2005	YES	YES	CD	YES	YES	YES	YES	YES	YES
Krowka.M J	1999	YES	YES	YES	YES	YES	YES	YES	YES	YES

Table S2. Subgroup analysis of all POPH patients.

Variables	Subgroup	Categories	No. of studies	MD (95% *CI*)	I^2 (%)	*P*-value across subgroups
mPAP	Age	≥55	8	-8.57 (-9.78 ~ -7.37)	0	0.56
		<55	17	-9.44 (-12.09 ~ -6.80)	62.50	
	Percentage of female	≥50%	14	-9.12(-12.43~-5.80)	68.50	0.79
		<50%	11	-8.65(-9.78~-7.52)	0	
	Sample size of PAH therapy	≥20	9	-8.84(-9.98~-7.70)	16.0	0.81
		<20	16	-9.23(-12.26~-6.21)	62.60	
	Drug treatment group	Prostacyclin group	9	-10.61(-14.93~-6.29)	75.70	0.42
		Non-prostacyclin group	16	-8.75(-10.07~-7.43)	7.70	
PVR	Age	≥55	8	-223.55(-283.65,-163.45)	84.0	0.47
		<55	17	-251.22(-295.26~-207.17)	19.68	
	Percentage of female	≥50%	14	-276.45(-325.37~-227.53)	0	0.05

		<50%	11	-211.26(-255.81~-166.70)	77.96	
	Sample size of PAH therapy	≥20	9	-229.06(-278.43~-179.69)	85.10	0.50
		<20	16	-251.33(-294.10~-208.56)	17.90	
	Drug treatment group	Prostacyclin group	9	-243.49(-299.88~-187.11)	50.40	0.85
		Non-prostacyclin group	16	-236.55(-278.24~-194.86)	81.0	
PAWP	Age	≥55	6	0.81(0.09~1.53)	34.0	0.34
		<55	8	1.82(-0.12~3.77)	78.1	
	Percentage of female	≥50%	6	2.29(-0.04~4.61)	79.9	0.22
		<50%	8	0.78(0.16~1.39)	13.1	
	Sample size of PAH therapy	≥20	8	0.80(0.21~1.40)	15.0	0.23
		<20	6	2.30(-0.10~4.71)	79.60	
	Drug treatment group	Prostacyclin group	3	-0.35(-2.44~1.73)	0	0.11

		Non-prostacyclin group	11	1.71(0.32~3.10)	78.40	
CO	Age	≥55	5	1.64(1.41~1.88)	0	1.00
		<55	14	1.64(1.05~2.23)	63.50	
	Percentage of female	≥50%	11	1.59(0.94~2.24)	66.10	0.91
		<50%	8	1.63(1.40~1.86)	3.0	
	Sample size of PAH therapy	≥20	5	1.63(1.40~1.86)	21.40	0.93
		<20	14	1.67(1.05~2.28)	58.90	
	Drug treatment group	Prostacyclin group	8	2.04(1.13~2.96)	49.80	0.40
		Non-prostacyclin group	11	1.60(1.13~2.07)	59.40	
Cardiac index	Age	≥55	6	0.66(0.42~0.91)	92.20	0.01
		<55	5	1.20(0.85~1.55)	47.40	
	Percentage of female	≥50%	6	0.97(0.58~1.36)	77.60	0.32

		<50%	5	0.72(0.41~1.03)	93.60	
	Sample size of PAH therapy	≥20	8	0.82(0.57~1.08)	90.90	0.99
		<20	3	0.83(0.11~1.55)	87.80	
RAP	Age	≥55	5	-1.59(-2.97~-0.21)	68.70	0.50
		<55	7	-0.77(-2.73~1.18)	53.70	
	Percentage of female	≥50%	8	-1.19(-3.07~0.68)	57.0	0.91
		<50%	4	-1.33(-2.72~0.06)	73.10	
	Sample size of PAH therapy	≥20	7	-1.63(-2.38~-0.89)	53.60	0.29
		<20	5	-0.12(-2.84~2.60)	58.10	

Note: 95%*CI*, 95%confidence interval; CO, cardiac output; mPAP, mean pulmonary artery pressure; PAH,Pulmonary hypertension; PAWP, Pulmonary wedge pressure; PVR, pulmonary vascular resistance; RAP, right atrial pressure.

The subgroup analysis of Cardiac index and RAP was carried out according to the classification of drug treatment. It was found that the number of studies was less than 3, so the subgroup analysis could not be completed.

Table S3. Subgroup analysis of patients with moderate and severe POPH.

Variables	Subgroup	Categories	No. of studies	MD (95% *CI*)	I^2 (%)	*P*-value across subgroups
mPAP	Age	≥55	3	-6.06 (-12.25 ~0.12)	0	0.28
		<55	14	-9.91 (-13.01 ~ -6.82)	67.0	
	Percentage of female	≥50%	12	-9.38(-13.26~-5.51)	71.80	0.85
		<50%	5	-8.90(-11.98~-5.82)	0	
	Drug treatment group	Prostacyclin group	8	-10.36(-15.27~-5.44)	78.60	0.78
		Non-prostacyclin group	9	-9.57(-12.08~-7.06)	16.40	
CO	Percentage of female	≥50%	10	1.54(0.79~2.29)	69.50	0.19
		<50%	5	2.35(1.41~3.29)	0	
	Drug treatment group	Prostacyclin group	7	2.02(0.98~3.05)	56.2	0.52
		Non-prostacyclin group	8	1.60(0.84~2.35)	63.6	

Note: mPAP, Mean pulmonary arterypressure; PVR, pulmonary vascular resistance; PAWP, Pulmonary wedge pressure; CO, Cardiac output; RAP, right atrial pressure; 95%CI, 95% confidence interval.

According to the above classification, the subgroup analysis of mPAP, PAWP, CO, Cardiac index

and RAP was carried out. Because the number of studies is less than 3, the subgroup analysis of PAWP, Cardiac index, RAP and some mPAP and CO groups cannot be completed.

Table S4. Meta-regression analyses of all POPH patients.

Independent variables	Dependent variables	Coefficient	Standard Error	95% Lower	95% Upper	z-value	*P*-value
mPAP	mPAP baseline	-0.08447	0.22602	-0.52745	0.35851	-0.37372	0.70861
	PVR baseline	0.00906	0.00469	-0.00013	0.01826	1.93178	0.05339
PVR	mPAP baseline	-10.91558	5.44474	-21.58707	-0.24410	-2.00480	0.04498
	PVR baseline	-0.11047	0.16253	-0.42902	0.20807	-0.67973	0.49668

Note: mPAP, mean pulmonary artery pressure; PVR, pulmonary vascular resistance.

Table S5. Meta-regression analyses of patients with moderate and severe POPH.

Independent variables	Dependent variables	Coefficient	Standard Error	95% Lower	95% Upper	z-value	*P*-value
mPAP	mPAP baseline	0.01033	0.27838	-0.53530	0.55595	0.03710	0.97040
	PVR baseline	0.01080	0.00553	-0.00004	0.02164	1.95238	0.05089
PVR	mPAP baseline	0.10255	0.06087	-0.01675	0.22186	1.68475	0.09204
	PVR baseline	0.00331	0.00100	0.00134	0.00527	3.29822	0.00097

Note: mPAP, Mean pulmonary arterypressure; PVR, pulmonary vascular resistance; 95%CI, 95% confidence interval.
